# Effect of acupuncture on neuroinflammation in animal models of Alzheimer’s disease: A preclinical systematic review and meta-analysis

**DOI:** 10.3389/fnagi.2023.1110087

**Published:** 2023-03-01

**Authors:** Zhi-Guo Wu, Ying-Jie Huang, Tun-Yi Wang, Chu-Yu Deng, Zhi-Rui Xu, Chun-Zhi Tang

**Affiliations:** Clinical Medical College of Acupuncture, Moxibustion and Rehabilitation, Guangzhou University of Chinese Medicine, Guangzhou, China

**Keywords:** Alzheimer’s disease, neuroinflammation, inflammatory cytokine, acupuncture, animal model, meta-analysis

## Abstract

**Background:**

Despite neuroinflammation being an important component of the pathology of Alzheimer’s disease (AD), effective therapies to alleviate neuroinflammation are still lacking. Many animal experiments in AD have found that acupuncture may ameliorate cognition by decreasing neuroinflammation and modulating cytokines, but its effects have not been systematically examined. We aimed to assess its efficacy on neuroinflammation in AD and to investigate the potential mechanisms.

**Materials and methods:**

The following databases were searched from inception until 24 August 2022: Web of Science, EMBASE, PubMed, the Cochrane Library, and China National Knowledge Infrastructure. Animal studies that reported the efficacy of acupuncture on neuroinflammation in AD were included. The SYRCLE Robt was utilized to evaluate methodological quality. Stata 17 was utilized to conduct a meta-analysis of cytokine levels and the results of the Morris water maze.

**Results:**

23 studies were included, with a total of 417 rats/mice. The overall quality of all included reports was medium. The results indicated that acupuncture significantly reduced the expressions of pro-inflammatory cytokines which included IL-1β [SMD = −3.50, 95% CI (−4.31, −2.69); *I*^2^ = 78.6%] (*P* < 0.05), TNF-α [SMD = −3.05, 95% CI (−3.86, −2.24); *I*^2^ = 69.6%] (*P* < 0.05), IL-6 [SMD = −3.22, 95% CI (−4.62, −1.81); *I*^2^ = 77.6%] and enhanced the expressions of anti-inflammatory cytokines including IL-4 [SMD = 2.77, 95% CI (1.95, 3.59); *I*^2^ = 33.9%] (*P* < 0.05), IL-10 [SMD = 1.84, 95% CI (1.20, 2.49); *I*^2^ = 41.0%] (*P* < 0.05) in an animal model of AD. Regarding the Morris water maze, compared to the control group, the acupuncture group showed a shorter escape latency [SMD = −2.23, 95% CI (−2.89, −1.57); *I*^2^ = 79.2%] (*P* < 0.05), longer duration in platform quadrant [SMD = 2.34, 95% CI (1.44, 3.23); *I*^2^ = 81.7%] (*P* < 0.05), and increased platform crossing number [SMD = 2.79, 95% CI (2.06, 3.53); *I*^2^ = 71.9%] (*P* < 0.05).

**Conclusion:**

Acupuncture may reduce neuroinflammation in AD by modulating cytokine expression. This modulation significantly improved cognitive function in animal models of AD.

**Systematic review registration:**

https://www.crd.york.ac.uk/PROSPERO/, identifier CRD42022354878.

## 1. Introduction

Alzheimer’s disease (AD) is a degenerative disease in the central nervous system (CNS), which is the commonest kind of dementia and happens frequently in individuals over sixty-five years of age. The typical symptoms of AD are progressive memory loss and cognitive impairment (Atri, 2019). Epidemiological investigations have revealed that there were 47 million AD patients in the world in 2019, and according to statistical data models, the prevalence is expected to increase more than trifold (about 131 million) in 2050 (Laird et al., 2009), placing a heavy economic burden on society and families. And it is urgent to find practical and effective treatment methods (Tiwari et al., 2019). Among the pathological features of AD, β-amyloid (Aβ) sedimentation is seen in extracellular plaques, and tau deposition is seen in intracellular neurofibrillary tangles (Knopman et al., 2021). Increasing evidence suggests that neuroinflammation caused by multiple reasons also plays a significant part in the pathogenesis of AD (Hane et al., 2017). Microglia and astrocytes engaged in immune execution in the CNS are continuously hyperactivated in AD and release cytokines and multiple inflammatory mediators (Heneka et al., 2015). There are two types of inflammatory cytokines, pro- and anti-inflammatory. Pro-inflammatory cytokines, such as TNF-α, IL-1β, IL-6, etc., produce neurotoxicity and enhance the inflammatory response. Conversely, IL-4 and IL-10 reduce inflammation and promote the repair of damaged tissues, which are considered anti-inflammatory cytokines (Rodríguez-Arellano et al., 2016). Studies have shown that TNF-α, IL-1β, and IL-6 are increased in the brains of AD sufferers (Bagyinszky et al., 2017) and produce secondary stimuli that promote microglia activation (Heppner et al., 2015). Chronic or uncontrolled activation of these inflammatory processes is detrimental by inducing neuronal injury or die-off (Halliday et al., 2000). Anti-neuroinflammatory strategies for AD include microglia modulators, astrocyte modulators, insulin resistance management, and microbiome therapy. However, there are no FDA-approved anti-neuroinflammatory therapies for AD (Yu et al., 2021b). Therefore, it is significant to find an anti-neuroinflammatory complementary and alternative therapy to delay disease progression and improve cognitive function in AD patients.

Acupuncture, a traditional Chinese medical method, is used in the complementary treatment of many diseases and is increasingly recognized for its multi-target modulation and fewer side effects. Studies suggested that drug therapy combined with acupuncture may be more effective for AD sufferers (Wang et al., 2020b). In addition, recent evidence suggests that acupuncture stimulates multiple neuroimmune pathways, ultimately acting immune cells *via* the release of crucial neurotransmitters and hormones to exert anti-inflammatory effects (Li et al., 2021b). Existing studies have recognized that acupuncture may play a potential anti-neuroinflammatory role in AD (Xu et al., 2020; Li et al., 2021a; Xie et al., 2021). However, a systematic understanding of how acupuncture inhibits neuroinflammation in AD is still lacking.

Therefore, we performed a meta-analysis on inflammatory cytokine and results of the Morris water maze in AD animal models and systematically investigated the possible mechanisms by which acupuncture modulates inflammatory cytokine expression to determine the efficacy of acupuncture on neuroinflammation in AD.

## 2. Materials and methods

We have registered this study with the PROSPERO international prospective register of systematic reviews (CRD42022354878). The latest Preferred Reporting Items for Systematic Reviews and Meta-Analyses (PRISMA) guidelines were utilized to conduct this study^1^ (Page et al., 2021).

### 2.1. Search strategy

The databases we searched included PubMed, EMBASE, the Cochrane Library, Web of Science, and China National Knowledge Infrastructure (CNKI) from inception until 24 August 2022. We have used the following terms: “electroacupuncture,” “acupuncture,” “senile dementia,” and “Alzheimer’s disease.” The detailed search strategies are shown in Supplementary Appendix 1. Also, reference lists of the selected articles in the original search results were reviewed to obtain more relevant studies. Studies in English and Chinese that reported the effects of acupuncture on neuroinflammation in animal models of AD were identified.

### 2.2. Eligibility criteria

Two researchers (Z-GW and Y-JH) jointly established inclusion and exclusion criteria for this review. The inclusion criteria for the review were as below: (1) a randomized controlled trial of an animal model of AD; (2) AD animal models, without strain, sex, and age restrictions, were successfully established in different ways; (3) manual acupuncture or electroacupuncture was the only intervention measure and no restrictions on acupoints, needling methods, treatment time, duration, and electroacupuncture parameters; (4) the levels of each inflammatory cytokine were included as the outcome measure. The exclusion criteria for the review were as follows: (1) studies that failed to establish an appropriate animal model, *ex vivo* studies, *in vitro* studies, and studies in humans or in sillico studies; (2) treatment group using other therapies or combination therapies; (3) studies where cytokine types did not meet our inclusion threshold nor did they measure our selected cytokines; (4) after the manual screening, other studies which didn’t meet our inclusion criteria were also excluded.

### 2.3. Data extraction

Two researchers (Z-GW and Y-JH) independently extracted the following data: (1) the year of publication and the first author’s name, (2) the basic details of the experimental animals (such as species, strain, sex, age, weight, and number), (3) method of establishing AD, (4) intervention characteristics (including the type of acupuncture, acupoints, treatment time, duration, electroacupuncture parameters, and the processing of the control group), and (5) outcomes (levels of each AD-associated neuroinflammatory cytokine and inflammatory mediator were extracted as primary outcome indicator, and behavioral tests of cognitive performance were extracted as the secondary outcome). If data were presented graphically only, GetData Graph Digitizer 2.26 was used to estimate the values from graphs.

### 2.4. Study risk of bias assessment

Two researchers (Z-GW and Y-JH) independently evaluated the risk of bias using the Systematic Review Center for Laboratory Animal Experimentation (SYRCLE) risk of bias tool (RoBT) (Hooijmans et al., 2014). This RoB tool can evaluate deviations in the below 10 domains: (1) sequence generation, (2) baseline characteristics, (3) allocation concealment, (4) random housing, (5) blinding of caregivers and investigators, (6) random outcome evaluation, (7) blinding of outcome assessment, (8) incomplete outcome data, (9) selective outcome reporting, and (10) other sources of bias. Two researchers negotiated to settle the dispute over the assessment, and if necessary, a third researcher (T-YW) could be contacted for arbitration.

### 2.5. Statistics

Data analysis was carried out using Stata 17 software. We regarded all outcomes to be continuous variables. When the results of each study were reported in different measures or units, standardized mean difference (SMD) is used as an effect-size Indices. We set the confidence interval (CI) to 95%. *P* < 0.05 was regarded as statistically significant. We evaluated heterogeneity by *Q*-test and *I*^2^-test. When *I*^2^ ≤ 50%, a fixed effects model was utilized. Otherwise, a random effects model is adopted. We analyzed publication bias by Egger’s test and funnel plots. Sensitivity analysis was implemented to test the stability and reliability of the results. For evaluating the impact of publication bias of the results, we applied the trim and fill method.

## 3. Results

### 3.1. Literature screening process

**FIGURE 1:**
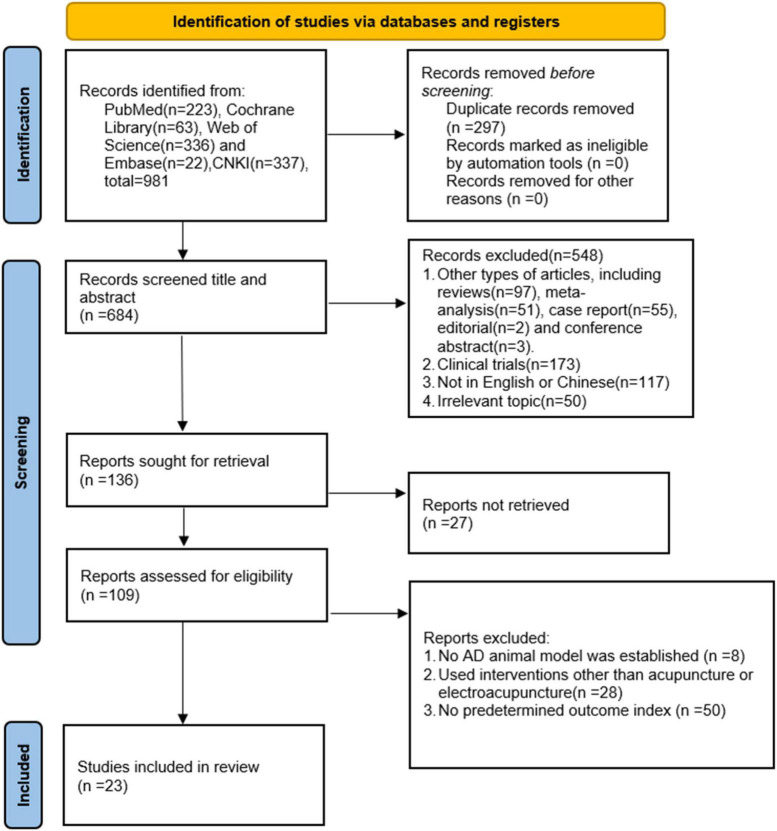
Literature screening process.

981 articles were retrieved from the database according to the search strategy set by two independent investigators (Z-GW and Y-JH). 297 repeated literature were eliminated. First, through reading titles and abstracts screening, other types of articles were eliminated: 97 reviews, 51 Meta-analysis, 55 case reports, 2 editorials, 3 conference abstracts, 173 clinical trials, 117 languages other than Chinese and English, and 50 unrelated topics. Then search reports were sought and 27 unsearched reports were excluded. Next, 86 articles were excluded by reading the full text for the following reasons: (1) No AD animal model was established8; (2) Used other interventions than acupuncture or electroacupuncture or acupuncture28; (3) No preoutcome index50, and finally 23 articles (Fang et al., 2013; Jiang et al., 2016, 2018, 2021; Ding et al., 2017; Wang et al., 2018b,^2019^, 2020a,c,^2021^; Tao et al., 2019, 2021; He et al., 2020a,b, 2021; Li et al., 2020, 2021a; Xu et al., 2020; Huang et al., 2021; Xie et al., 2021; Yang et al., 2021; Zhang et al., 2021; Liao et al., 2022) were included in our study (Figure 1).

### 3.2. Literature characteristics

23 studies with a total of 417 mice were included, 209 mice in the treatment group, and 208 mice in the control group. These included 11 studies (Ding et al., 2017; Jiang et al., 2018, 2021; Li et al., 2020, 2021a; Wang et al., 2020a,^2021^; Xu et al., 2020; He et al., 2021; Xie et al., 2021; Zhang et al., 2021) in English and 12 studies (Fang et al., 2013; Jiang et al., 2016; Wang et al., 2018b,^2019^, 2020c; Tao et al., 2019, 2021; He et al., 2020a,b; Huang et al., 2021; Yang et al., 2021; Liao et al., 2022) in Chinese. These studies were published from 2013 to 2022 (Supplementary Table 1).

Acupoint selection and combination: All studies mentioned the use of acupoints, 1 study (Zhang et al., 2021) used 4 acupoints, 8 studies (Fang et al., 2013; Jiang et al., 2018; Wang et al., 2018b, 2020a,c; Xu et al., 2020; Liao et al., 2022) used 3 acupoints, 14 studies (Jiang et al., 2016, 2021; Ding et al., 2017; Tao et al., 2019, 2021; Wang et al., 2019, 2021; He et al., 2020a,b, 2021; Li et al., 2020, 2021a; Huang et al., 2021; Yang et al., 2021) used 2 acupoints and 1 study (Xie et al., 2021) used 1 acupoint. We performed statistics on the frequency of acupoint use. The higher frequency of acupoint use was (GV20) 18 times, (BL23) 7 times, (ST36) 6 times, (GV29) 5 times, (DU20) 2 times, and (KI3) 2 times. A total of six studies used GV20 + BL23, and GV20 + ST36, which are the most common combinations of acupoint use, followed by four studies that used GV20 + GV29. In terms of acupuncture stimulation maneuvers, which were mainly divided into MA, and EA. MA was utilized in 3 researches (Jiang et al., 2016; Ding et al., 2017; Zhang et al., 2021) and EA in 20 researches (Fang et al., 2013; Jiang et al., 2018, 2021; Wang et al., 2018b,^2019^, 2020a,c,^2021^; Tao et al., 2019, 2021; He et al., 2020a,b, 2021; Li et al., 2020, 2021a; Xu et al., 2020; Huang et al., 2021; Xie et al., 2021; Yang et al., 2021; Liao et al., 2022; Supplementary Table 1).

Electroacupuncture wave pattern frequency and frequency intensity: Of the 15 studies (Fang et al., 2013; Jiang et al., 2018, 2021; Wang et al., 2018b, 2019, 2020a; Tao et al., 2019, 2021; He et al., 2020a,b, 2021; Li et al., 2020; Huang et al., 2021; Yang et al., 2021; Liao et al., 2022) that mentioned electroacupuncture wave pattern, 11 studies (Fang et al., 2013; Wang et al., 2018b,^2019^; Tao et al., 2019, 2021; He et al., 2020a,b, 2021; Huang et al., 2021; Yang et al., 2021; Liao et al., 2022) used Continuous wave, 3 study (Jiang et al., 2018, 2021; Wang et al., 2020a) used sparse wave, and 1 study (Li et al., 2020) used disperse-dense wave. 20 studies (Fang et al., 2013; Jiang et al., 2018, 2021; Wang et al., 2018b,^2019^, 2020a,c, 2021; Tao et al., 2019, 2021; He et al., 2020a,b, 2021; Li et al., 2020, 2021a; Xu et al., 2020; Huang et al., 2021; Xie et al., 2021; Yang et al., 2021; Liao et al., 2022) mentioned the frequency of electroacupuncture, ranging from 1/20 Hz to 50 Hz, including 1/20 Hz for 1 study (Li et al., 2020), 2 Hz for 10 studies (Fang et al., 2013; Jiang et al., 2018, 2021; Wang et al., 2018b,2020a,c; Xu et al., 2020; Li et al., 2021a; Yang et al., 2021; Liao et al., 2022), 5 Hz for 3 studies (Tao et al., 2019, 2021; Wang et al., 2019), 15 Hz for 1 study, 20 Hz for 1 study (Xie et al., 2021), and 50 Hz for 4 studies (He et al., 2020a,b, 2021; Huang et al., 2021). 20 studies (Fang et al., 2013; Jiang et al., 2018, 2021; Wang et al., 2018b,^2019^, 2020a,c,^2021^; Tao et al., 2019, 2021; He et al., 2020a,b, 2021; Li et al., 2020, 2021a; Xu et al., 2020; Huang et al., 2021; Xie et al., 2021; Yang et al., 2021; Liao et al., 2022) mentioned the intensity of electroacupuncture, 2 of which used 0.1 mA (Jiang et al., 2021; Yang et al., 2021) 0.2 of which used 0.6 mA (Jiang et al., 2018; Wang et al., 2020a), 10 of which used 1 mA (Fang et al., 2013; Wang et al., 2018b; He et al., 2020a,b, 2021; Li et al., 2020, 2021a; Xu et al., 2020; Huang et al., 2021; Liao et al., 2022) 1 of which used 1.5 mA (Wang et al., 2021), 1 of which used 2 mA (Wang et al., 2020c), 1 of which used 20 mA (Xie et al., 2021), and the intensity of 3 studies (Tao et al., 2019, 2021; Wang et al., 2019) was based on Setting the current strength as the standard for leg shaking (Supplementary Table 1).

Animal species and modeling methods: All studies described the species of experimental animals, including 10 studies (Fang et al., 2013; Jiang et al., 2016; Wang et al., 2018b, 2020c, 2021; He et al., 2020a,b, 2021; Huang et al., 2021; Xie et al., 2021) in SD rats, 6 studies (Ding et al., 2017; Jiang et al., 2018, 2021; Wang et al., 2020a; Yang et al., 2021; Zhang et al., 2021) in SAMP8 mice, 3 studies (Wang et al., 2018b,^2019^; Tao et al., 2019) in Wistar rats, and 1 study (Li et al., 2020) in APP/PS1 mice. In different studies, AD models were established using different methods, and a total of 13 studies (Fang et al., 2013; Jiang et al., 2016; Wang et al., 2018b,^2019^, 2020c,^2021^; Tao et al., 2019, 2021; He et al., 2020a,b, 2021; Huang et al., 2021; Xie et al., 2021) mentioned modeling methods, of which 4 studies (He et al., 2020a,b, 2021; Huang et al., 2021) used intraperitoneal injection, 8 studies (Fang et al., 2013; Wang et al., 2018b,^2019^, 2020c,^2021^; Tao et al., 2019, 2021; Xie et al., 2021) used Hippocampal injection, and 1 study (Jiang et al., 2016) used Lateral ventricular injection of STZ (Supplementary Table 1).

Outcome Measures: IL-1β, TNF-α, IL6, IL4, IL10, Water maze test (escape latency, the duration in platform quadrant, platform crossing number) 0.21 researches (Fang et al., 2013; Jiang et al., 2016, 2018, 2021; Ding et al., 2017; Wang et al., 2018b,^2019^, 2020c,^2021^; He et al., 2020a,b, 2021; Li et al., 2020, 2021a; Xu et al., 2020; Huang et al., 2021; Tao et al., 2021; Xie et al., 2021; Yang et al., 2021; Zhang et al., 2021; Liao et al., 2022) mentioned IL-1β; 14 studies (Wang et al., 2018b,^2019^, 2020a,c,^2021^; He et al., 2020b,^2021^; Huang et al., 2021; Jiang et al., 2021; Li et al., 2021a; Tao et al., 2021; Xie et al., 2021; Yang et al., 2021; Liao et al., 2022) mentioned TNF-α; 8 studies (He et al., 2020b,^2021^; Li et al., 2020; Huang et al., 2021; Jiang et al., 2021; Wang et al., 2021; Xie et al., 2021; Yang et al., 2021) mentioned IL6; 3 studies (Wang et al., 2019; Tao et al., 2021; Xie et al., 2021) mentioned IL4; 4 studies (Tao et al., 2019; Wang et al., 2019; Xu et al., 2020; Xie et al., 2021) mentioned IL10. 16 studies (Jiang et al., 2016, 2021; Ding et al., 2017; He et al., 2020a,b, 2021; Li et al., 2020, 2021a; Wang et al., 2020a,c, 2021; Xu et al., 2020; Huang et al., 2021; Yang et al., 2021; Zhang et al., 2021; Liao et al., 2022) involved escape latency; 10 studies (Ding et al., 2017; He et al., 2020a,b, 2021; Xu et al., 2020; Huang et al., 2021; Jiang et al., 2021; Li et al., 2021a; Zhang et al., 2021; Liao et al., 2022) involved the duration in platform quadrant; 13 studies (Jiang et al., 2016; Ding et al., 2017; He et al., 2020a,b; Li et al., 2020, 2021a; Wang et al., 2020a,c, 2021; Xu et al., 2020; Xie et al., 2021; Zhang et al., 2021; Liao et al., 2022) mentioned platform crossing number (Supplementary Table 1).

### 3.3. Risk of bias

**FIGURE 2:**
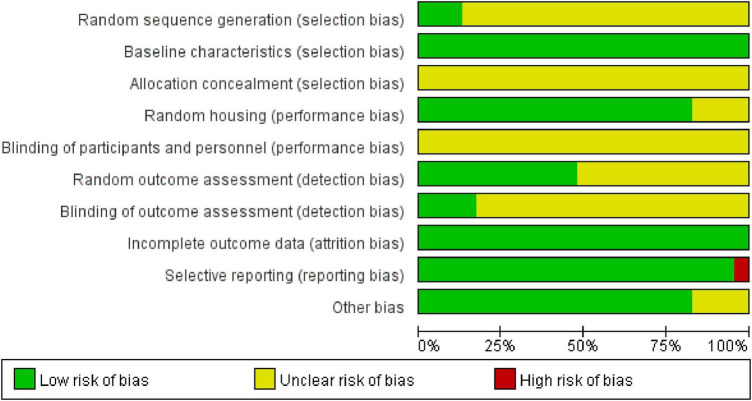
Risk of bias assessment for included studies.

All included reports were of medium quality overall. According to the SYRCLE risk assessment tool, the evaluation results were as follows: all studies were randomized, 3 (He et al., 2020a,b; Liao et al., 2022) of which explicitly mentioned the animal randomization method, all of which were random number methods, and the other 20 studies did not specifically describe the way of randomization. None of the animals included in the studies had statistically significant differences in baseline data, but none mentioned allocation concealment. Random housing was not explicitly stated in any of the studies, but 19 studies (Jiang et al., 2016, 2018, 2021; Ding et al., 2017; Wang et al., 2018b,^2019^, ^2021^; Tao et al., 2019, 2021; He et al., 2020a,^2021^; Li et al., 2020, 2021a; Xu et al., 2020; Huang et al., 2021; Xie et al., 2021; Yang et al., 2021; Zhang et al., 2021; Liao et al., 2022) described animal housing conditions and 4 studies (Fang et al., 2013; He et al., 2020b; Wang et al., 2020a,c) did not specifically describe animal housing conditions. Because the intervention in these studies was acupuncture, the investigators could not implement blinding. Of the 23 studies evaluated for results, 12 studies (Jiang et al., 2016, 2021; Tao et al., 2019, 2021; Wang et al., 2019, 2020a,c; Li et al., 2020; Xu et al., 2020; Yang et al., 2021; Zhang et al., 2021; Liao et al., 2022) randomly selected animals for evaluation of results and the remaining 11 studies did not clearly describe whether animals were randomly selected for evaluation of results. 4 studies (Li et al., 2020, 2021a; Wang et al., 2021; Zhang et al., 2021) mentioned blinding clearly in the result statistics, while the others did not mention the implementation of blinding clearly. 1 study (Wang et al., 2019) reported selective results and the literature data were complete. In terms of other sources of bias, 4 studies (Fang et al., 2013; Wang et al., 2020a,c; He et al., 2021)did not explicitly mention specific treatment measures in the control group (Figure 2 and Supplementary Figure 1).

### 3.4. IL-1β meta-analysis

**FIGURE 3:**
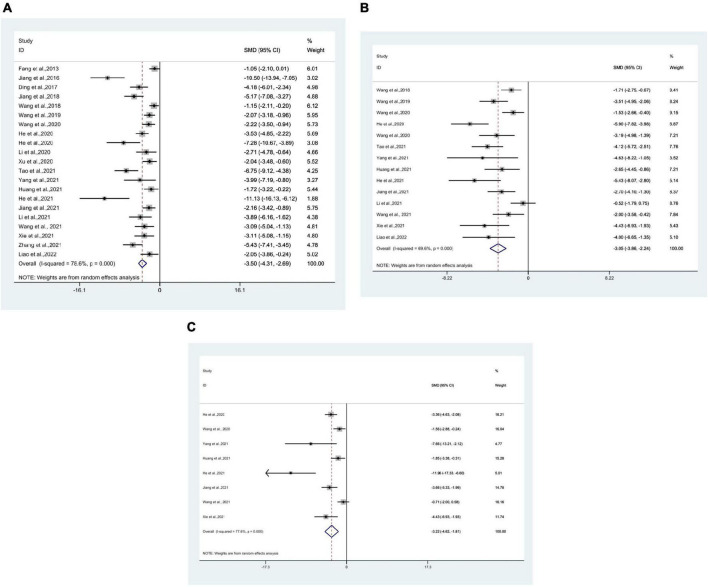
Forest plots. **(A)** Forest plot showing the effects of acupuncture on the levels of IL-1β in animal models of Alzheimer’s disease (AD); **(B)** forest plot showing the effects of acupuncture on the levels of TNF-α in animal models of AD; **(C)** forest plot showing the effects of acupuncture on the levels of IL-6 in animal models of AD.

21 researches (Fang et al., 2013; Jiang et al., 2016, 2018, 2021; Ding et al., 2017; Wang et al., 2018b,^2019^, 2020c,^2021^; He et al., 2020a,b, 2021; Li et al., 2020, 2021a; Xu et al., 2020; Huang et al., 2021; Tao et al., 2021; Xie et al., 2021; Yang et al., 2021; Zhang et al., 2021; Liao et al., 2022) used IL-1β as an outcome measure, with 307 mice in total. Meta-analysis revealed that IL-1β was significantly lower in the test group than in the control group [SMD = −3.50, 95% CI (−4.31, −2.69); *I*^2^ = 78.6%], and there was a statistically significant difference (*P* < 0.05) (Figure 3A).

### 3.5. TNF-α meta-analysis

14 studies (Wang et al., 2018b,^2019^, 2020a,c,^2021^; He et al., 2020b,^2021^; Huang et al., 2021; Jiang et al., 2021; Li et al., 2021a; Tao et al., 2021; Xie et al., 2021; Yang et al., 2021; Liao et al., 2022) used TNF-α as an outcome measure, with 194 mice in total. Meta-analysis revealed that in the test group TNF-α was significantly lower than in the control group [SMD = −3.05, 95% CI (−3.86, −2.24); *I*^2^ = 69.6%], and statistically significant difference was found (*P* < 0.05) (Figure 3B).

### 3.6. IL-6 meta-analysis

8 studies (He et al., 2020b,^2021^; Wang et al., 2020a,^2021^; Huang et al., 2021; Jiang et al., 2021; Xie et al., 2021; Yang et al., 2021) using IL-6 as the outcome measure, with 100 mice in total. Meta-analysis indicated that IL-6 was significantly lower in the test group compared to the control group [SMD = −3.22, 95% CI (−4.62, −1.81); *I*^2^ = 77.6%], and statistics showed a significant difference (*P* < 0.05) (Figure 3C).

### 3.7. IL-4 meta-analysis

**FIGURE 4:**
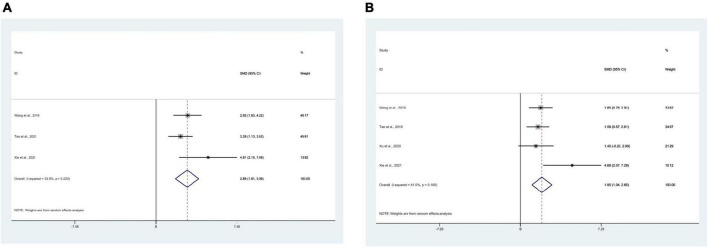
Forest plots. **(A)** Forest plot showing the effects of acupuncture on the levels of IL-4 in animal models of Alzheimer’s disease (AD); **(B)** forest plot showing the effects of acupuncture on the levels of IL-10 in animal models of AD.

3 studies (Wang et al., 2019; Tao et al., 2021; Xie et al., 2021) using IL-4 as the outcome measure, with 50 mice in total. Meta-analysis indicated that IL-4 was significantly higher in the test group compared to the control group [SMD = 2.77, 95% CI (1.95, 3.59); *I*^2^ = 33.9%], and statistics indicated a significant difference (*P* < 0.05) (Figure 4A).

### 3.8. IL-10 meta-analysis

4 studies (Tao et al., 2019; Wang et al., 2019; Xu et al., 2020; Xie et al., 2021) using IL-10 as the outcome measure, with 58 mice in total. Meta-analysis displayed that IL-10 was significantly higher in the test group compared to the control group [SMD = 1.84, 95% CI (1.20, 2.49); *I*^2^ = 41.0%], and the statistically significant difference was found (*P* < 0.05) (Figure 4B).

### 3.9. Meta-analysis of water maze test

#### 3.9.1. Escape latency

**FIGURE 5:**
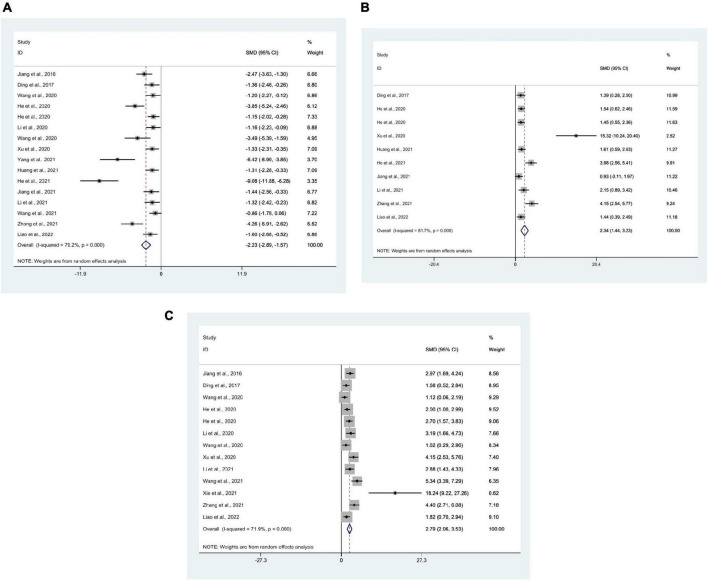
Forest plots. **(A)** Forest plot showing the effects of acupuncture on the escape latency in the Morris water maze in animal models of Alzheimer’s disease (AD); **(B)** forest plot showing the effects of acupuncture on the duration in platform quadrant in the Morris water maze in animal models of AD; **(C)** forest plot showing the effects of acupuncture on the platform crossing number in the Morris water maze in animal models of AD.

16 studies (Jiang et al., 2016, 2021; Ding et al., 2017; He et al., 2020a,b, 2021; Li et al., 2020, 2021a; Wang et al., 2020a,c, 2021; Xu et al., 2020; Huang et al., 2021; Yang et al., 2021; Zhang et al., 2021; Liao et al., 2022) used mean escape latency as the outcome measure, with 299 mice in total. Meta-analysis displayed that the escape latency of the test group, compared with the control group, was significantly lower [SMD = −2.23, 95% CI (−2.89, −1.57); *I*^2^ = 79.2%], and there was a statistically significant difference between the groups (*P* < 0.05) (Figure 5A).

#### 3.9.2. The duration in platform quadrant

10 studies (Ding et al., 2017; He et al., 2020a,b, 2021; Xu et al., 2020; Huang et al., 2021; Jiang et al., 2021; Li et al., 2021a; Zhang et al., 2021; Liao et al., 2022) used the duration in platform quadrant as an outcome measure, with 198 mice in total. Meta-analysis manifested that compared with the control group, the duration in platform quadrant in the test group was significantly higher [SMD = 2.34, 95% CI (1.44, 3.23); *I*^2^ = 81.7%], and there was a statistically significant difference between the groups (*P* < 0.05) (Figure 5B).

#### 3.9.3. Platform crossing number

There were 13 studies (Jiang et al., 2016; Ding et al., 2017; He et al., 2020a,b; Li et al., 2020, 2021a; Wang et al., 2020a,c, 2021; Xu et al., 2020; Xie et al., 2021; Zhang et al., 2021; Liao et al., 2022) that used platform crossing number as an outcome measure, with 233 mice in total. Meta-analysis manifested that the platform crossing number in the test group, compared with the control group, was significantly more [SMD = 2.79, 95% CI (2.06, 3.53); *I*^2^ = 71.9%], and statistics indicated a significant difference between the groups (*P* < 0.05) (Figure 5C).

### 3.10. Subgroup meta-analysis

#### 3.10.1. IL-1β

A subgroup Meta-analysis was performed based on the acupuncture types (EA and MA). The results of the subgroup Meta-analysis revealed that compared to the control group, IL-1β in the MA group was significantly lower [SMD = −6.35, 95% CI (−9.35, −3.36); *I*^2^ = 80.2%]; IL-1β in the EA group compared to the control group, was significantly lower [SMD = −3.02, 95% CI (−3.76, −2.27); *I*^2^ = 72.5%], and statistics indicated a significant difference between the groups (*P* < 0.05). The subgroup Meta-analysis was performed for the course of treatment, and the results displayed that compared to the control group, IL-1β at 1.5–3, 4–6, and 8–16 w in the test group was significantly lower, and statistics indicated a significant difference between the groups (*P* < 0.05). This indicated that different acupuncture treatments were not statistically significant on IL-1β among studies. At the same time, different modeling methods, strain and age of AD animals were analyzed by subgroup meta-analysis. The results of subgroup meta-analysis showed that IL-1β in the test group was lower than that in the control group (*P* < 0.05), indicating that different modeling methods, strain and age had no statistically significant effect on IL-1β (Supplementary Table 2).

#### 3.10.2. TNF-α

Because the acupuncture methods used in studies involving TNF-α are electroacupuncture, no subgroup of acupuncture methods can be made. A subgroup Meta-analysis was performed for the course of treatment, and the subgroup Meta-analysis indicated that TNF-α at 1.5–3, 4–6, and 8 w in the test group, compared to the control group, was significantly lower, and statistics indicated a significant difference between the groups (*P* < 0.05). This indicated that different acupuncture treatments were not statistically significant on TNF-α among studies. Subgroup meta-analysis of different modeling methods, strains, ages in AD animals showed that TNF-α in the test group was lower than that in the control group (*P* < 0.05), indicating that different modeling methods, strains, ages had no statistically significant effect on TNF-α (Supplementary Table 2).

#### 3.10.3. IL-6

Because the acupuncture methods used in studies involving IL-6 were electroacupuncture, no subgroup of acupuncture methods could be done. Subgroup meta-analysis of the course of treatment revealed that compared to the control group, IL-6 at 2–3, 4–6, and 8 w in the test group was significantly lower, and there was a statistically significant difference between the groups (*P* < 0.05). This indicated that different acupuncture treatments were not statistically significant on IL-6 among studies. Subgroup meta-analysis of strain and age showed that IL-6 in the test group was lower than that in the control group (*P* < 0.05), indicating that strain and age had no statistically significant effect on IL-6. Subgroup analysis of different modeling methods showed that IL-6 in the test group using hippocampal injection was not statistically significant compared with the control group (*P* > 0.05), which may be due to the fact that only two articles of AD animals were modeled using hippocampal injection (Supplementary Table 2).

#### 3.10.4. Escape latency

We performed a subgroup Meta-analysis for the acupuncture methods. The subgroup Meta-analysis revealed that the escape latency in the MA group, compared to the control group, was significantly lower [SMD = −2.59, 95% CI (−4.10, −1.08); *I*^2^ = 76.0%]; the escape latency in the EA group, compared to the control group, was significantly lower [SMD = −2.16, 95% CI (−2.90, −1.41); *I*^2^ = 80.1%], and the statistically significant difference was found (*P* < 0.05). Subgroup Meta-analysis was also performed for the course of treatment, and the results indicated that the escape latency of 1.5–3, 4–6, and 8–16 w in the test group, compared to the control group, was significantly lower, and the statistically significant difference was found (*P* < 0.05). This indicated that different acupuncture treatments were not statistically significant on escape latency among studies. Subgroup meta-analysis was performed for different modeling methods, strains, and ages in AD animals, and the results of subgroup analysis showed that escape latency in the test group was lower than that in the control group (*P* < 0.05), indicating that different modeling methods, strains, and ages had no statistically significant effect on escape latency (Supplementary Table 2).

#### 3.10.5. The duration in platform quadrant

We performed a subgroup Meta-analysis for the acupuncture methods. The results indicated that compared to the control group, the target quadrant exploration time of the MA group was significantly higher [SMD = 2.71, 95% CI (0.01, 5.41); *I*^2^ = 86.9%]; the duration in platform quadrant of EA group, compared to the control group, was significantly higher [SMD = 2.27, 95% CI (1.25, 3.28); *I*^2^ = 82.8%], and the statistically significant difference was found (*P* < 0.05). Subgroup Meta-analysis was performed for the course of treatment, and the subgroup Meta-analysis indicated that compared to the control group, the target quadrant exploration time of 2–3, 4–6, and 8 w in the test group was significantly higher, and the statistically significant difference was found (*P* < 0.05). This indicated that different acupuncture treatments were not statistically significant on the duration in platform quadrant among studies. Subgroup meta-analysis was performed for different modeling methods, strains, and ages in AD animals, and the results of subgroup analysis showed that the duration in platform quadrant in the test group was higher than that in the control group (*P* < 0.05), indicating that different modeling methods, strains, and ages had no statistically significant effect on the duration in platform quadrant (Supplementary Table 2).

#### 3.10.6. Platform crossing number

We performed a subgroup analysis of the acupuncture methods. Subgroup Meta-analysis manifested that compared to the control group, the platform crossing number in the MA group was significantly more [SMD = 2.92, 95% CI (1.45, 4.38); *I*^2^ = 71.5%]; the platform crossing number in the EA group, compared to the control group, was significantly more [SMD = 2.78, 95% CI (1.89, 3.67); *I*^2^ = 74.5%], and the statistically significant difference was found (*P* < 0.05). The subgroup Meta-analysis was performed for the course of treatment, and the subgroup Meta-analysis manifested that platform crossing number at 1.5–3, 4–5, and 8 w in the test group, compared to the control group, was significantly more and statistics indicated a significant difference (*P* < 0.05). This indicated that different acupuncture treatments were not statistically significant on platform crossing number among studies. Subgroup meta-analysis was performed for different modeling methods, strains, and ages in AD animals, and the results of subgroup analysis showed that the platform crossing number in the test group was higher than that in the control group (*P* < 0.05), indicating that different modeling methods, strains, and ages had no statistically significant effect on platform crossing number (Supplementary Table 2).

### 3.11. Publication bias

The effect size of IL-1β, TNF-α, escape latency, the duration in platform quadrant, platform crossing number were used as abscissae and standard error was used as ordinate for comparative–corrected funnel plots, as shown in the Supplementary Figure 2. Points of different colors in the inverted funnel plot indicate direct comparisons of different interventions, and the number of points of the same color indicates the number of pairwise comparisons in the study. According to the results, some scatter involving IL-1β, TNF-α, escape latency, the duration in platform quadrant, platform crossing number were basically outside the range of inverted funnel plot, and Egger’s test showed that *P* < 0.05, which means that there may be publication bias.

### 3.12. Sensitivity analysis

The sensitivity analysis was performed to test Meta’s stability and identify sources of heterogeneity. Results from sensitivity analysis indicated that all outcomes were stable (*P* < 0.05) (Supplementary Figure 3). For evaluating the impact of publication bias of the results (IL-1β, TNF-α, escape latency, the duration in platform quadrant, platform crossing number), we applied the trim and fill method. The results indicated the robustness of these results was not significantly affected by publication bias (Table 1).

**TABLE 1:** Results from Egger’s test and trim and fill analysis.

Outcomes	Egger’s test	Before trim and fill			After trim and fill		
	***P*-value**	***P*-value**	**Est (F/R)**	**No. studies**	***P*-value**	**Est (F/R)**	**No. studies**
Escape latency	0.000	0.000	−1.738/−2.233	16	0.000	−1.596/−1.810	18
The duration in platform quadrant	0.000	0.000	1.847/2.337	10	0.000	1.847/2.337	10
Platform crossing number	0.000	0.000	2.472/2.794	13	0.000	2.347/2.527	15
IL-1β	0.000	0.000	−2.662/−3.499	21	0.000	−2.005/−2.102	30
TNF-α	0.004	0.000	−2.604/−3.050	14	0.000	−2.283/−2.476	18

Est, total effect sizes; F/R, fixed effect model/random-effects model; No., number.

## 4. Discussion

International research on traditional Chinese medicine for the treatment of AD has focused on Chinese herbs [e.g., Morinda officinalis How (Zhang and Zhang, 2022), Astragali radix (Dong et al., 2022)] or active ingredients of Chinese herbs [e.g., polysaccharides (Zhang et al., 2022), ginsenoside (Wu et al., 2022a), evodiamine (Pang et al., 2022), Huperzine A (Yan et al., 2022)], herbal prescriptions [e.g., Yi-Gan San (Yang et al., 2023), Yuan-Zhi Decoction (Wu et al., 2022b)], special therapies [e.g., Wuqinxi exercise (Luo et al., 2022), Tai Chi Quan (Wang et al., 2022)], and acupuncture (Cai et al., 2019; Yu et al., 2021a; Zheng et al., 2021; Hao et al., 2022) to improve memory and cognitive impairment in AD models, involving various mechanisms such as regulation of β-amyloid and tau proteins, anti-oxidation, anti-neuroinflammation, modulation of autophagy and many other mechanisms. Among these, acupuncture is considered to be a highly promising alternative therapy for AD. With a growing number of studies suggest a strong relationship between neuroinflammation and AD (Sardi et al., 2011; Calsolaro and Edison, 2016; Spangenberg and Green, 2017; Fakhoury, 2018). In recent years some cutting-edge researchers have endeavored to investigate whether acupuncture can ameliorate the neuroinflammation of AD. Many researchers have found in animal studies that acupuncture, an alternative therapy, can reduce neuroinflammation in AD (Xu et al., 2020; Li et al., 2021a; Xie et al., 2021). However, to my knowledge, no study has systematically investigated the effects of acupuncture on inflammatory cytokine expression in the course of AD. This preclinical systematic review and meta-analysis included 23 researches (417 experimental animals in total) to ascertain whether acupuncture can improve cognitive function in animal models of AD by analyzing behavioral indicators and to further assess the effectiveness and potential mechanism of acupuncture on inflammatory cytokine expression in AD.

In AD pathology, Aβ bind to pattern recognition receptors (such as CD36, TLR4, and TLR6) on microglia and astrocyte and trigger an innate immune response (Heneka et al., 2015), which drive microglia to a pro-inflammatory phenotype and to release pro-inflammatory cytokines (Colonna and Butovsky, 2017). The pro-inflammatory phenotype of microglia induces inflammation and neurotoxicity, and persistent inflammatory stimulation exacerbates disease progression and severity. However, microglia activation in the central nervous system is heterogeneous, which also has an anti-inflammatory phenotype that secretes anti-inflammatory cytokines that induce anti-inflammation and neuroprotection (Guo et al., 2022). Studies have shown that cytokines such as peripheral and central TGF-β, IL-1β, IL-6 and TNF-α, are elevated in the cerebrospinal fluid and peripheral blood of AD patients, but IL-4 and IL-10 are reduced or do not change significantly (Swardfager et al., 2010). Interestingly, Microglial activation is both characterized and modulated by cytokines. Studies using preclinical animal models have shown that abnormal protein accumulation, glial cell initiation, and cytokine responses are interrelated and progressively amplified processes in AD (Perry and Holmes, 2014). Cytokines are associated with nearly every aspect of neuroinflammation. Our study indicated that acupuncture reduced the expression of pro-inflammatory cytokines (IL-1β, TNF-α, and IL-6) in AD animal models. On the contrary, acupuncture promoted the expression of anti-inflammatory cytokines (IL-4 and IL-10) in AD animal models. In addition, in the Morris water maze, acupuncture treatment indicated beneficial effects on cognitive function in the AD animal model, as evidenced by a shorter escape latency, longer duration in platform quadrant, and increased platform crossing number. This suggested that acupuncture may reduce neuroinflammation in the AD animal models *via* modulating cytokine secretion, which in turn improves cognitive function in AD animal models. Meanwhile, this finding supported the work of other studies in this area linking neuroinflammation to AD.

**FIGURE 6:**
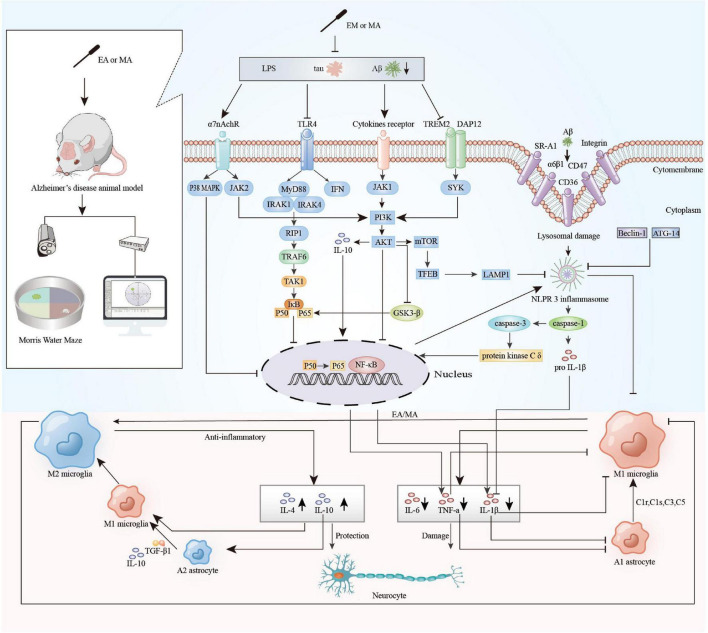
The main mechanisms of acupuncture to regulate neuroinflammation in animal models of Alzheimer’s disease (AD). EA, electroacupuncture; MA, manual acupuncture; LPS, lipopolysaccharide; Tau, microtubule-associated protein tau; Aβ, beta-amyloid; α7nAChR, alpha-7 nicotinic acetylcholine receptor; TLR4, toll-Like receptor 4; TREM2, triggering receptor expressed on myeloid cells-2; DAP12, DNAX-activating protein 12; SR-A1, scavenger receptor A1; α6β1, integrin α6β1; P38 MAPK, P38 mitogen activated protein kinases; JAK2, janus kinase 2; MyD88, myeloid differentiation factor 88; IFN, interferon; JAK1, janus kinase 1; SYK, spleen tyrosine kinase; IRAK1, interleukin-1 receptor-associated kinase1; IRAK4, interleukin-1 receptor-associated kinase 4; RIP1, receptor-interacting protein 1; TRAF6, tumor necrosis factor receptor-associated factor 6; TAK1, transforming growth factor β activated kinase 1; Iκβ, inhibitor κB kinase β; NF-κB, nuclear factor κ-B; PI3K, phosphatidylinositol 3-kinase; Akt, protein kinase B; GSK-3β, glycogen synthase kinase-3β; mTOR, mammalian target of rapamycin; TFEB, transcription factor EB; LAMP, lysosome-associated membrane protein 1; ATG-14, autophagy protein 14; Beclin 1, autophagic protein Beclin 1; NLRP3 inflammasome, nod-like receptor protein 3 inflammasome; TGF-β1, transforming growth factor-β1; C1r, complement 1r; C1s, complement 1s; C3, complement 3; C5, complement 5; TNF-α, tumor necrosis factor-a; IL-1β, interleukin 1β; IL-4, interleukin 4; IL-6, interleukin 6; IL-10, interleukin 10.

Cytokines are predominantly produced by macrophages/microglia and lymphocytes and mediate cellular function, cell signaling, and neuroimmune activity (Arango Duque and Descoteaux, 2014). As mentioned above, cytokines play a pivotal part in the neuroinflammation of AD. IL-1β increases sAPPα secretion *via* MEK 1/2, JNK-activated α-secretase cleavage (Ma et al., 2005) and metalloprotease (ADAM)-17/TNF-α converting enzyme (TACE) pathway (Tachida et al., 2008) and elevates mRNAs encoding α-synuclein, βAPP, tau, and MAPK-p38 (Griffin et al., 2006). In addition, IL-1β induces microglia proliferation and upregulates astrocyte expression through paracrine effects, while upregulating IL-6, TNF-α, CSF-1, and TGF-β1 (Buxbaum et al., 1992). TNF-α induces APP mRNA expression *via* NFκB activation (Sommer et al., 2009) and upregulates sAPPβ by increasing β- and γ-secretase (Liao et al., 2004; Wang et al., 2018a). A model study found that pretreatment with a TNF-α inhibitor prevented synaptic deficiency induced by increased secretion of TNF-α in the hippocampus (Cavanagh et al., 2016). AD cortical senile plaques display strong IL-6 immunoreactivity (Bauer et al., 1991), which is functionally related to alpha-2-macroglobulin (Strauss et al., 1992). IL-6 also stimulates the synthesis of the APP (Altstiel and Sperber, 1991) and facilitates tau phosphorylation (Wang et al., 2018a). Conversely, IL-4 can attenuate Aβ-induced LTP deficiency, and the secretion of proinflammatory IL-1β in rat hippocampus (Lyons et al., 2007). IL-4 may induce selective o-Abeta (1–42) clearance to reduce AD pathology (Shimizu et al., 2008). IL-4 induction may also contribute to neuroprotection *via* the IRS-1/-2 signaling pathway in the hippocampus (Kiyota et al., 2010). IL-10 can reduce excessive inflammatory responses, but its role is controversial in AD. AAV2/1 infection of hippocampal neurons resulted in sustained expression of IL-10 without its leakage into the blood, reduced astro/microgliosis, enhanced plasma Aβ peptide levels, and enhanced neurogenesis (Kiyota et al., 2012). But Paramita et al. found that IL-10 may increase ApoE expression and promote its co-deposition with Aβ *via* altering immune protein homeostasis in AD mice (Chakrabarty et al., 2015). The results of our systematic review suggested that the main mechanisms by which acupuncture reduces neuroinflammation in AD include:(1) Aβ is an important driver of neuroinflammation, which drive microglia to activating and to release inflammatory cytokines. Acupuncture inhibits the cumulation of Aβ, accelerates the clearance of senile plaques and toxic substances by improving microcirculation in brain and enhances brain glucose metabolism to reduce the inflammatory response (Jiang et al., 2016; Xu et al., 2020). (2) Hyperactivation of microglia and astrocytes is a major source of neuroinflammation in AD. Activated immune cells release cytokines and chemokines that exacerbate the inflammatory response of AD. Acupuncture upregulates the expression of cholinergic nerve-associated protein such as ChAT, α7nAChR to attenuate glial cell overexpression, regulates microglial polarization, downregulates the M1 phenotype microglia and upregulates the M2 phenotype microglia (Li et al., 2020; Wang et al., 2020a; Huang et al., 2021; Xie et al., 2021). (3) Inflammasomes are important mediators of Aβ-induced caspase-1 activation and secretion of pro-inflammatory and neurotoxic cytokines. Acupuncture can decrease the expression of NLRP3 and NLRP1 inflammasome-related proteins and regulate the NLRP3/Caspase-1 pathway negatively (Ding et al., 2017; Jiang et al., 2018; He et al., 2020a; Li et al., 2021a; Zhang et al., 2021); (4) TLR4, distributed in microglia and astrocytes, is an important pattern recognition receptor involved in the neuroinflammatory response in AD. TLR4/NF-κB signaling pathway-induced inflammatory cascade response fosters immune cell activation, cytokine release and neuronal damage. Acupuncture down-regulates TLR4/NF-κB signaling pathway to reduce neuroinflammation (He et al., 2021; Tao et al., 2021). (5) Increased intestinal permeability and blood-brain barrier disruption induced by dysbiosis of the intestinal microbiota may be important factors in intracerebral inflammation in patients with AD (Shabbir et al., 2021). Acupuncture regulates the community structure of gut microbiota and upregulate the expression of Claudin-5, ZO-1 to inhibit the peripheral inflammatory response and the neuroinflammatory response in hippocampus (Jiang et al., 2021; Liao et al., 2022; Figure 6). These findings, although preliminary, suggest that acupuncture does have a positive effect on neuroinflammation in AD. How to find the targets that has the dominant role from multiple targets in the modulatory effects of acupuncture is an important question for future research.

We found that the combined outcomes of the included studies showed high heterogeneity. The design of animal studies is usually more exploratory and heterogeneous than that of clinical trials. We performed subgroup analyses according to animal species, types of acupuncture, acupuncture schedules and used the exclusion method, but no significant sources of heterogeneity were found. Subsequently, we performed meta-analyses and sensitivity analyses using random effects models.

We have noted that many aspects of the current study design are flawed. Firstly, the internal validity of animal studies refers to the scientific robustness of study design, implementation, analysis, and reporting (Pound and Ritskes-Hoitinga, 2018). We found that included studies have the problem of internal validity. For example, acupuncturists were unable to administer blinding, which may have led to an overestimation of the beneficial effects of acupuncture. In addition, the description of the included studies regarding the random assignment and blinded assessment of outcomes is unclear, which leads us to worry that there are serious internal validity issues. Secondly, no existing animal model can perfectly simulate all pathological features of AD in human beings, and there are no standard operating procedures for the identification of AD animal models at present (Zhang et al., 2020). But there are certain differences in behavioral and pathological characteristics between the various models. All studies included five different strains of rodent models. This could be a potential source of heterogeneity. Advanced age is the biggest risk factor for AD (van der Lee et al., 2018). In addition, women are more likely to develop AD than men (Knopman et al., 2021). However, some of the studies used low-month-old animal models, and few studies focused on the sex of the animal models. These failures of animal models to accurately represent human disease and clinical context are sometimes described as failures of construct validity, which is often considered a subset of external validity (van der Worp et al., 2010). Thirdly, because treatment in Traditional Chinese Medicine is based on the differentiation of syndromes (Zhong et al., 2014), it is difficult to standardize the parameters of acupuncture, such as acupoints and electroacupuncture parameters, among different patients in clinical treatment, and it is also difficult to standardize the parameter settings applied in animal experiments, which may increase the irreproducibility of treatment effects based on animal experiments and undermined external validity of the study. The translation of findings from animals to humans can only occur reliably if preclinical animal studies are both internally and externally valid (Pound and Ritskes-Hoitinga, 2018). Therefore, more emphasis should be placed on internal and external validity in future animal experiments on AD. In addition, we recommend defining a standard operating procedure for modeling evaluation and emphasizing the influence of risk factors such as age and gender in future studies. Lastly, we think that further research should be undertaken to examine the effectiveness of different acupoints and acupuncture parameters on AD.

Our study has the following limitations: (1) There may be potential language bias in our study because acupuncture-related research exists in other language countries (e.g., Korea, Japan) but our search results included only English and Chinese literature. (2) Despite we do my best efforts to search all eligible literature, the number of studies included in the analysis was low (especially the limited number of studies investigating the regulation of anti-inflammatory cytokines by acupuncture). (3) The methodological descriptions of some studies were unclear, resulting in limited reliability of the risk of bias evaluation. (4) The high heterogeneity among the included literature and the inability to fully interpret it may reduce the certainty of effect estimates. (5) There was some degree of publication bias because negative results are unlikely to be published. (6) Our study only addressed a fraction of the cytokines that play a role in AD, due to the limited current exploration of related areas.

## 5. Conclusion

Taken together, this systematic review suggests that acupuncture may improve cognitive impairment in AD by modulating cytokine expression. The main potential mechanisms include that acupuncture modulates Aβ, glial cell, NLRP3, and NLRP1 inflammasome-related proteins expression, down-regulates TLR4/NF-κB signaling pathway, and regulates gut microbiota. Further studies are required to explore in depth acupuncture’s therapeutic effects on neuroinflammation in AD. We hope that our study provides some useful references for other researchers.

## Data availability statement

The original contributions presented in this study are included in this article/Supplementary material, further inquiries can be directed to the corresponding author.

## Author contributions

Z-GW, Y-JH, and C-ZT conceived and designed the study. Z-GW and Y-JH searched the database and extracted the data. Z-GW, Y-JH, and T-YW analyzed the data and wrote the first draft of the manuscript. C-YD, Z-RX, and C-ZT revised the manuscript. All authors read and approved the final manuscript.

## Footnotes

1
http://www.prisma-statement.org

## References

[B1] Altstiel L. D. Sperber K. (1991). Cytokines in Alzheimer’s disease. *Prog. Neuro-psychopharmacol. Biol. Psychiatry* 15 481–495. 10.1016/0278-5846(91)90023-t 1749826

[B2] Arango Duque G. Descoteaux A. (2014). Macrophage cytokines: involvement in immunity and infectious diseases. *Front. Immunol.* 5:491. 10.3389/fimmu.2014.00491 25339958 PMC4188125

[B3] Atri A. (2019). The Alzheimer’s disease clinical spectrum: diagnosis and management. *Med. Clin. North Am.* 103 263–293. 10.1016/j.mcna.2018.10.009 30704681

[B4] Bagyinszky E. Giau V. V. Shim K. Suk K. An S. S. A. Kim S. (2017). Role of inflammatory molecules in the Alzheimer’s disease progression and diagnosis. *J. Neurol. Sci.* 376 242–254. 10.1016/j.jns.2017.03.031 28431620

[B5] Bauer J. Strauss S. Schreiter-Gasser U. Ganter U. Schlegel P. Witt I. (1991). Interleukin-6 and alpha-2-macroglobulin indicate an acute-phase state in Alzheimer’s disease cortices. *FEBS Lett.* 285 111–114. 1712317 10.1016/0014-5793(91)80737-n

[B6] Buxbaum J. D. Oishi M. Chen H. I. Pinkas-Kramarski R. Jaffe E. A. Gandy S. E. (1992). Cholinergic agonists and interleukin 1 regulate processing and secretion of the Alzheimer beta/A4 amyloid protein precursor. *Proc. Natl. Acad. Sci. U S A.* 89 10075–10078. 10.1073/pnas.89.21.10075 1359534 PMC50280

[B7] Cai M. Lee J.-H. Yang E. J. (2019). Electroacupuncture attenuates cognition impairment via anti-neuroinflammation in an Alzheimer’s disease animal model. *J. Neuroinflamm.* 16:264. 10.1186/s12974-019-1665-3 31836020 PMC6909515

[B8] Calsolaro V. Edison P. (2016). Neuroinflammation in Alzheimer’s disease: current evidence and future directions. *Alzheimer’s Dement.* 12 719–732. 10.1016/j.jalz.2016.02.010 27179961

[B9] Cavanagh C. Tse Y. C. Nguyen H.-B. Krantic S. Breitner J. C. S. Quirion R. (2016). Inhibiting tumor necrosis factor-α before amyloidosis prevents synaptic deficits in an Alzheimer’s disease model. *Neurobiol. Aging* 47 41–49. 10.1016/j.neurobiolaging.2016.07.009 27552480

[B10] Chakrabarty P. Li A. Ceballos-Diaz C. Eddy J. A. Funk C. C. Moore B. (2015). IL-10 alters immunoproteostasis in APP mice, increasing plaque burden and worsening cognitive behavior. *Neuron* 85 519–533. 10.1016/j.neuron.2014.11.020 25619653 PMC4320003

[B11] Colonna M. Butovsky O. (2017). Microglia function in the central nervous system during health and neurodegeneration. *Ann. Rev. Immunol.* 35 441–468. 10.1146/annurev-immunol-051116-052358 28226226 PMC8167938

[B12] Ding N. Jiang J. Lu M. Hu J. Xu Y. Liu X. (2017). Manual acupuncture suppresses the expression of proinflammatory proteins associated with the NLRP3 inflammasome in the hippocampus of SAMP8 mice. *Evidence-Based Complementary Alternat. Med.* 2017:3435891. 10.1155/2017/3435891 28904553 PMC5585617

[B13] Dong Q. Li Z. Zhang Q. Hu Y. Liang H. Xiong L. (2022). Astragalus mongholicus Bunge (Fabaceae): bioactive compounds and potential therapeutic mechanisms against Alzheimer’s disease. *Front. Pharmacol.* 13:924429. 10.3389/fphar.2022.924429 35837291 PMC9273815

[B14] Fakhoury M. (2018). Microglia and astrocytes in Alzheimer’s disease: implications for therapy. *Curr. Neuropharmacol.* 16 508–518. 10.2174/1570159X15666170720095240 28730967 PMC5997862

[B15] Fang J. Zhu S. Zhang Y. Wang F. Zhu Q. (2013). Effect of electroacupuncture on expressiong of phosphory lated P38MAPK and IL-1 βin frontal lobe and hippocampus in rats with Alzheimer’s disease. *Acupuncture Res.* 38 35–39. 10.13702/j.1000-0607.2013.01.007 23650798

[B16] Griffin W. S. T. Liu L. Li Y. Mrak R. E. Barger S. W. (2006). Interleukin-1 mediates Alzheimer and Lewy body pathologies. *J. Neuroinflamm.* 3:5. 10.1186/1742-2094-3-5 PMC1435743 16542445

[B17] Guo S. Wang H. Yin Y. (2022). Microglia polarization from M1 to M2 in neurodegenerative diseases. *Front. Aging Neurosci.* 14:815347. 10.3389/fnagi.2022.815347 35250543 PMC8888930

[B18] Halliday G. Robinson S. R. Shepherd C. Kril J. (2000). Alzheimer’s disease and inflammation: a review of cellular and therapeutic mechanisms. *Clin. Exp. Pharmacol. Physiol.* 27 1–8. 10696521 10.1046/j.1440-1681.2000.03200.x

[B19] Hane F. T. Lee B. Y. Leonenko Z. (2017). Recent progress in Alzheimer’s disease research, part 1: pathology. *J. Alzheimer’s Dis. JAD* 57 1–28. 10.3233/JAD-160882 28222507

[B20] Hao X. Ding N. Zhang Y. Yang Y. Zhao Y. Zhao J. (2022). Benign regulation of the gut microbiota: the possible mechanism through which the beneficial effects of manual acupuncture on cognitive ability and intestinal mucosal barrier function occur in APP/PS1 mice. *Front. Neurosci.* 16:960026. 10.3389/fnins.2022.960026 35992924 PMC9382294

[B21] He C. Huang C. Chen H. Yu C. Wang X. Jiang D. (2020a). Effect of pre-acupuncture on learning-memory ability and related protein of NLRP3 inflammasome in hippocampus in Alzheimer’s disease like rats. *Chinese Acupuncture* 40 1323–1327. 10.13703/j.0255-2930.20191012-k0004 33415876

[B22] He C. Huang C. Chen H. Yu C. Zheng Q. Wang Y. (2020b). Effect of pre-acupuncture on learning and memory ability and TLR4/NF-κB signaling pathway in AD like rats. *J. Pract. Med.* 36 2510–2514.

[B23] He C. Huang Z.-S. Yu C.-C. Wang X.-S. Jiang T. Wu M. (2021). Preventive electroacupuncture ameliorates D-galactose-induced Alzheimer’s disease-like inflammation and memory deficits, probably via modulating the microbiota-gut-brain axis. *Iran. J. Basic Med. Sci.* 24 341–348. 10.22038/ijbms.2021.49147.11256 33995945 PMC8087854

[B24] Heneka M. T. Carson M. J. El Khoury J. Landreth G. E. Brosseron F. Feinstein D. L. (2015). Neuroinflammation in Alzheimer’s disease. *Lancet Neurol.* 14 388–405. 10.1016/S1474-4422(15)70016-5 25792098 PMC5909703

[B25] Heppner F. L. Ransohoff R. M. Becher B. (2015). Immune attack: the role of inflammation in Alzheimer disease. *Nat. Rev. Neurosci.* 16 358–372. 10.1038/nrn3880 25991443

[B26] Hooijmans C. R. Rovers M. M. de Vries R. B. M. Leenaars M. Ritskes-Hoitinga M. Langendam M. W. (2014). SYRCLE’s risk of bias tool for animal studies. *BMC Med. Res. Methodol.* 14:43. 10.1186/1471-2288-14-43 24667063 PMC4230647

[B27] Huang C. He C. Chen H. Yu C. Wang X. Jiang T. (2021). Effect of pre-electro-acupuncture on cholinergic nerve-related proteins on learning and memory abilities and inflammation in the brain of AD-like rats. *Chinese J. Gerontol.* 41 562–567.

[B28] Jiang J. Ding N. Wang K. Li Z. (2018). Electroacupuncture could influence the expression of IL-1 beta and NLRP3 inflammasome in hippocampus of Alzheimer’s disease animal model. *Evidence-Based Complementary Altern. Med.* 2018:8296824. 10.1155/2018/8296824 30105072 PMC6076968

[B29] Jiang J. Liu H. Wang Z. Tian H. Wang S. Yang J. (2021). Electroacupuncture could balance the gut microbiota and improve the learning and memory abilities of Alzheimer’s disease animal model. *PLoS One* 16:e0259530. 10.1371/journal.pone.0259530 34748592 PMC8575259

[B30] Jiang M. Liang J. Zhang Y. Wang J. Hao J. Wang M. (2016). Effect of acupuncture of “Siguan” acupoint on learning and memory and β amyloid 42, interleukin-1 β and interleukin-2 in Alzheimer’s disease rats. *Acupuncture Res.* 41 113–118. 10.13702/j.1000-0607.2016.02.004

[B31] Kiyota T. Ingraham K. L. Swan R. J. Jacobsen M. T. Andrews S. J. Ikezu T. (2012). AAV serotype 2/1-mediated gene delivery of anti-inflammatory interleukin-10 enhances neurogenesis and cognitive function in APP+PS1 mice. *Gene Therapy* 19 724–733. 10.1038/gt.2011.126 21918553 PMC3241853

[B32] Kiyota T. Okuyama S. Swan R. J. Jacobsen M. T. Gendelman H. E. Ikezu T. (2010). CNS expression of anti-inflammatory cytokine interleukin-4 attenuates Alzheimer’s disease-like pathogenesis in APP+PS1 bigenic mice. *FASEB J.* 24 3093–3102. 10.1096/fj.10-155317 20371618 PMC2909296

[B33] Knopman D. S. Amieva H. Petersen R. C. Chételat G. Holtzman D. M. Hyman B. T. (2021). Alzheimer disease. *Nat. Rev. Dis. Primers* 7:33. 10.1038/s41572-021-00269-y PMC8574196 33986301

[B34] Laird M. H. W. Rhee S. H. Perkins D. J. Medvedev A. E. Piao W. Fenton M. J. (2009). TLR4/MyD88/PI3K interactions regulate TLR4 signaling. *J. Leukocyte Biol.* 85 966–977. 10.1189/jlb.1208763 19289601 PMC2698589

[B35] Li K. Shi G. Zhao Y. Chen Y. Gao J. Yao L. (2021a). Electroacupuncture ameliorates neuroinflammation-mediated cognitive deficits through inhibition of NLRP3 in Presenilin1/2 conditional double knockout mice. *Neural Plast* 2021:8814616. 10.1155/2021/8814616 33505459 PMC7806385

[B36] Li N. Guo Y. Gong Y. Zhang Y. Fan W. Yao K. (2021b). The anti-inflammatory actions and mechanisms of acupuncture from acupoint to target organs via neuro-immune regulation. *J. Inflamm. Res.* 14 7191–7224. 10.2147/JIR.S341581 34992414 PMC8710088

[B37] Li L. Li L. Zhang J. Huang S. Liu W. Wang Z. (2020). Disease stage-associated alterations in learning and memory through the electroacupuncture modulation of the cortical microglial M1/M2 polarization in mice with Alzheimer’s disease. *Neural Plast* 2020:8836173. 10.1155/2020/8836173 PMC7474773 32908486

[B38] Liao D. Pang F. Zhou M. Li Y. Yang Y. Guo X. (2022). Effect of electroacupubcture on cognitive impairment in APP/PS1 mice based on TLR4/NF-κB/NLRP3 pathway. *Acupuncture Res.* 47 565–572. 10.13702/j.1000-0607.20210604 35880271

[B39] Liao Y.-F. Wang B.-J. Cheng H.-T. Kuo L.-H. Wolfe M. S. (2004). Tumor necrosis factor-alpha, interleukin-1beta, and interferon-gamma stimulate gamma-secretase-mediated cleavage of amyloid precursor protein through a JNK-dependent MAPK pathway. *J. Biol. Chem.* 279 49523–49532. 10.1074/jbc.M402034200 15347683

[B40] Luo S.-S. Chen L. Wang G.-B. Wang Y.-G. Su X.-Y. (2022). Effects of long-term Wuqinxi exercise on working memory in older adults with mild cognitive impairment. *Eur. Geriatric Med.* 13 1327–1333. 10.1007/s41999-022-00709-2 36327046

[B41] Lyons A. Downer E. J. Crotty S. Nolan Y. M. Mills K. H. G. Lynch M. A. (2007). CD200 ligand receptor interaction modulates microglial activation in vivo and in vitro: a role for IL-4. *J. Neurosci.* 27 8309–8313. 10.1523/JNEUROSCI.1781-07.2007 17670977 PMC6673084

[B42] Ma G. Chen S. Wang X. Ba M. Yang H. Lu G. (2005). Short-term interleukin-1(beta) increases the release of secreted APP(alpha) via MEK1/2-dependent and JNK-dependent alpha-secretase cleavage in neuroglioma U251 cells. *J. Neurosci. Res.* 80 683–692. 10.1002/jnr.20515 15880353

[B43] Page M. J. McKenzie J. E. Bossuyt P. M. Boutron I. Hoffmann T. C. Mulrow C. D. (2021). The PRISMA 2020 statement: an updated guideline for reporting systematic reviews. *BMJ (Clinical Research ed)* 372:n71. 10.1136/bmj.n71 33782057 PMC8005924

[B44] Pang S. Li S. Cheng H. Luo Z. Qi X. Guan F. (2022). Discovery of an evodiamine derivative for PI3K/AKT/GSK3β pathway activation and AD pathology improvement in mouse models. *Front. Mol. Neurosci.* 15:1025066. 10.3389/fnmol.2022.1025066 36698780 PMC9868638

[B45] Perry V. H. Holmes C. (2014). Microglial priming in neurodegenerative disease. *Nat. Rev. Neurol.* 10 217–224. 10.1038/nrneurol.2014.38 24638131

[B46] Pound P. Ritskes-Hoitinga M. (2018). Is it possible to overcome issues of external validity in preclinical animal research? Why most animal models are bound to fail. *J. Trans. Med.* 16:304. 10.1186/s12967-018-1678-1 30404629 PMC6223056

[B47] Rodríguez-Arellano J. J. Parpura V. Zorec R. Verkhratsky A. (2016). Astrocytes in physiological aging and Alzheimer’s disease. *Neuroscience* 323 170–182. 10.1016/j.neuroscience.2015.01.007 25595973

[B48] Sardi F. Fassina L. Venturini L. Inguscio M. Guerriero F. Rolfo E. (2011). Alzheimer’s disease, autoimmunity and inflammation. the good, the bad and the ugly. *Autoimmun. Rev.* 11 149–153. 10.1016/j.autrev.2011.09.005 21996556

[B49] Shabbir U. Arshad M. S. Sameen A. Oh D.-H. (2021). Crosstalk between gut and brain in Alzheimer’s disease: the role of gut microbiota modulation strategies. *Nutrients* 13:690. 10.3390/nu13020690 33669988 PMC7924846

[B50] Shimizu E. Kawahara K. Kajizono M. Sawada M. Nakayama H. (2008). IL-4-induced selective clearance of oligomeric beta-amyloid peptide(1-42) by rat primary type 2 microglia. *J. Immunol.* 181 6503–6513. 10.4049/jimmunol.181.9.6503 18941241

[B51] Sommer G. Kralisch S. Lipfert J. Weise S. Krause K. Jessnitzer B. (2009). Amyloid precursor protein expression is induced by tumor necrosis factor alpha in 3T3-L1 adipocytes. *J. Cell. Biochem.* 108 1418–1422. 10.1002/jcb.22382 19862700

[B52] Spangenberg E. E. Green K. N. (2017). Inflammation in Alzheimer’s disease: lessons learned from microglia-depletion models. *Brain Behav. Immun.* 61 1–11. 10.1016/j.bbi.2016.07.003 27395435 PMC5218993

[B53] Strauss S. Bauer J. Ganter U. Jonas U. Berger M. Volk B. (1992). Detection of interleukin-6 and alpha 2-macroglobulin immunoreactivity in cortex and hippocampus of Alzheimer’s disease patients. *Lab. Invest.* 66 223–230. 1370967

[B54] Swardfager W. Lanctôt K. Rothenburg L. Wong A. Cappell J. Herrmann N. (2010). A meta-analysis of cytokines in Alzheimer’s disease. *Biol. Psychiatry* 68 930–941. 10.1016/j.biopsych.2010.06.012 20692646

[B55] Tachida Y. Nakagawa K. Saito T. Saido T. C. Honda T. Saito Y. (2008). Interleukin-1 beta up-regulates TACE to enhance alpha-cleavage of APP in neurons: resulting decrease in Abeta production. *J. Neurochem.* 104 1387–1393. 10.1111/j.1471-4159.2007.05127.x 18021299

[B56] Tao Y. Du Y. Tian Q. Wang J. Sun G. Shen F. (2019). Effects of electroacupuncture on the expression and co-localization of hippocampal dentate astrocytes and IL-10 in AD rats. *Chinese J. Gerontol.* 39 874–878.

[B57] Tao Y. Du Y. Wang J. Sun G. Xiao J. (2021). Role of TLR4 /NF -KB signaling pathway and effect of electroacupuncture on Alzheimer’s disease inflammatory response. *China J. Trad. Chinese Med.* 39 168–171. 10.13193/j.issn.1673-7717.2021.01.042

[B58] Tiwari S. Atluri V. Kaushik A. Yndart A. Nair M. (2019). Alzheimer’s disease: pathogenesis, diagnostics, and therapeutics. *Int. J. Nanomed.* 14 5541–5554. 10.2147/IJN.S200490 31410002 PMC6650620

[B59] van der Lee S. J. Wolters F. J. Ikram M. K. Hofman A. Ikram M. A. Amin N. (2018). The effect of APOE and other common genetic variants on the onset of Alzheimer’s disease and dementia: a community-based cohort study. *Lancet Neurol.* 17 434–444. 10.1016/S1474-4422(18)30053-X 29555425

[B60] van der Worp H. B. Howells D. W. Sena E. S. Porritt M. J. Rewell S. O’Collins V. (2010). Can animal models of disease reliably inform human studies? *PLoS Med.* 7:e1000245. 10.1371/journal.pmed.1000245 20361020 PMC2846855

[B61] Wang M.-M. Miao D. Cao X.-P. Tan L. Tan L. (2018a). Innate immune activation in Alzheimer’s disease. *Ann. Trans. Med.* 6:177. 10.21037/atm.2018.04.20 PMC5994517 29951499

[B62] Wang X. Liu R. Shen C. Zhou H. Sun G. Shen F. (2018b). Effect of low-frequency electroacupuncture on the expression of inflammatory factors in rats with Alzheimer’s disease. *Chinese J. Gerontol.* 38 1701–1703.

[B63] Wang R. Zhou H. Wang Y.-C. Chang X.-L. Wang X.-Q. (2022). Benefits of Tai Chi Quan on neurodegenerative diseases: a systematic review. *Ageing Res. Rev.* 82:101741. 10.1016/j.arr.2022.101741 36220604

[B64] Wang X. Li Z. Li C. Wang Y. Yu S. Ren L. (2020a). Electroacupuncture with Bushen Jiannao improves cognitive deficits in senescence-accelerated mouse prone 8 mice by inhibiting neuroinflammation. *J. Tradit. Chin. Med.* 40 812–819. 10.19852/j.cnki.jtcm.2020.05.011 33000582

[B65] Wang Y.-Y. Yu S.-F. Xue H.-Y. Li Y. Zhao C. Jin Y.-H. (2020b). Effectiveness and safety of acupuncture for the treatment of Alzheimer’s disease: a systematic review and meta-analysis. *Front. Aging Neurosci.* 12:98. 10.3389/fnagi.2020.00098 PMC7218057 32435187

[B66] Wang Y. Wu X. Tang C. Xu Y. Wang J. Xu J. (2020c). The efficacy and mechanism of different acupoints in Alzheimer’s rats. *Acupuncture Res.* 45 617–622. 10.13702/j.1000-0607.190887 32869570

[B67] Wang Y. Tao Y. Sun G. Xiao J. Du Y. (2019). Effect of electroacupuncture on inflammatory factors in serum and brain of AD rats induced by Aβ1∼42. *Chinese J. Gerontol.* 39 1921–1927.

[B68] Wang Y. Zheng A. Yang H. Wang Q. Ren B. Guo T. (2021). “Olfactory three-needle” acupuncture enhances synaptic function in A beta(1-42)-induced Alzheimer’s disease via activating PI3K/AKT/GSK-3 beta signaling pathway. *J. Integrat. Neurosci.* 20 55–65. 10.31083/j.jin.2021.01.224 33834691

[B69] Wu J.-J. Yang Y. Wan Y. Xia J. Xu J.-F. Zhang L. (2022a). New insights into the role and mechanisms of ginsenoside Rg1 in the management of Alzheimer’s disease. *Biomed. Pharmacotherapy = Biomed. Pharmacotherapie* 152:113207. 10.1016/j.biopha.2022.113207 35667236

[B70] Wu Q. Li X. Jiang X.-W. Yao D. Zhou L.-J. Xu Z.-H. (2022b). Yuan-Zhi decoction in the treatment of Alzheimer’s disease: an integrated approach based on chemical profiling, network pharmacology, molecular docking and experimental evaluation. *Front. Pharmacol.* 13:893244. 10.3389/fphar.2022.893244 36091836 PMC9451491

[B71] Xie L. Liu Y. Zhang N. Li C. Sandhu A. F. Williams G. III (2021). Electroacupuncture improves M2 microglia polarization and glia anti-inflammation of hippocampus in Alzheimer’s disease. *Front. Neurosci.* 15:689629. 10.3389/fnins.2021.689629 34646113 PMC8502881

[B72] Xu A. Tang Y. Zeng Q. Wang X. Tian H. Zhou Y. (2020). Electroacupuncture enhances cognition by promoting brain glucose metabolism and inhibiting inflammation in the app/ps1 mouse model of Alzheimer’s disease: a pilot study. *J. Alzheimers Dis.* 77 387–400. 10.3233/jad-200242 32741819

[B73] Yan Y.-P. Chen J.-Y. Lu J.-H. (2022). Disease-Modifying activity of huperzine a on Alzheimer’s disease: evidence from preclinical studies on rodent models. *Int. J. Mol. Sci.* 23:15238. 10.3390/ijms232315238 36499562 PMC9738397

[B74] Yang J. Jiang J. Tian H. Wang Z. Ren Q. Liu H. (2021). Effect of electroacupuncture on learning-memory ability and expression of IL-1, IL-6 and TNF-α hippocampus and spleen in mice with Alzheimer’s disease mice. *Acupuncture Res.* 46 353–361. 10.13702/j.1000-0607.200980 34085456

[B75] Yang S.-Y. Lin Z.-X. Xian Y.-F. Zhang H.-M. Xu H.-X. (2023). Traditional uses, chemical compounds, pharmacological activities and clinical studies on the traditional Chinese prescription Yi-Gan San. *J. Ethnopharmacol.* 302(Pt A):115859. 10.1016/j.jep.2022.115859 36280017

[B76] Yu C.-C. He C. Du Y.-J. Gao S. Lin Y.-F. Wang S.-Q. (2021a). Preventive electroacupuncture reduces cognitive deficits in a rat model of D-galactose-induced aging. *Neural Regenerat. Res.* 16 916–923. 10.4103/1673-5374.297090 33229729 PMC8178792

[B77] Yu T.-W. Lane H.-Y. Lin C.-H. (2021b). Novel therapeutic approaches for Alzheimer’s disease: an updated review. *Int. J. Mol. Sci.* 22:8208. 10.3390/ijms22158208 34360973 PMC8348485

[B78] Zhang L. Chen C. Mak M. S. Lu J. Wu Z. Chen Q. (2020). Advance of sporadic Alzheimer’s disease animal models. *Med. Res. Rev.* 40 431–458. 10.1002/med.21624 31328804

[B79] Zhang T. Guan B. Tan S. Zhu H. Ren H. D. Li R. (2021). Bushen huoxue acupuncture inhibits NLRP1 inflammasome-mediated neuronal pyroptosis in SAMP8 mouse model of Alzheimer’s disease. *Neuropsychiatric Dis. Treatment* 17 339–346. 10.2147/ndt.S279304 PMC7872899 33574670

[B80] Zhang X. Lin L. Li H. Xia W. Liu Q. Zhou X. (2022). Update on new trend and progress of the mechanism of polysaccharides in the intervention of Alzheimer’s disease, based on the new understanding of relevant theories: a review. *Int. J. Biol. Macromol.* 218 720–738. 10.1016/j.ijbiomac.2022.07.158 35902016

[B81] Zhang Y. Zhang M. (2022). Neuroprotective effects of morinda officinalis how: anti-inflammatory and antioxidant roles in Alzheimer’s disease. *Front. Aging Neurosci.* 14:963041. 10.3389/fnagi.2022.963041 36158563 PMC9493036

[B82] Zheng X. Lin W. Jiang Y. Lu K. Wei W. Huo Q. (2021). Electroacupuncture ameliorates beta-amyloid pathology and cognitive impairment in Alzheimer disease via a novel mechanism involving activation of TFEB (transcription factor EB). *Autophagy* 17 3833–3847. 10.1080/15548627.2021.1886720 33622188 PMC8632298

[B83] Zhong Y. Liu B. Qu H. Xie Q. (2014). Methodological challenges to human medical study. *Front. Med.* 8:328–336. 10.1007/s11684-014-0359-6 25159994

