# Supplementary material for: Effect of acupuncture on neuroinflammation in animal models of Alzheimer’s disease: A preclinical systematic review and meta-analysis
Source: Front Aging Neurosci. 2023 Mar 1;15:1110087. doi: 10.3389/fnagi.2023.1110087 (PMC10014858; doi:10.3389/fnagi.2023.1110087)
Supplement: Supplementary file 1 [file Table_1.DOCX]

Supplementary Material

**Appendix 1.** Search strategies for four English databases.

**Supplementary Figure 1.** Risk of bias assessment.

**Supplementary Figure 2.** Funnel plot.

**Supplementary Figure 3.** The sensitivity analysis.

**Appendix 1.** Search strategies for four English databases.

1. **PubMed**

(((((((((((((((((((((((((((((((((((("Alzheimer Disease"[Mesh]) OR (Alzheimer Dementia[Title/Abstract])) OR (Alzheimer Dementias[Title/Abstract])) OR (Dementia, Alzheimer[Title/Abstract])) OR (Alzheimer's Disease[Title/Abstract])) OR (Dementia, Senile[Title/Abstract])) OR (Senile Dementia[Title/Abstract])) OR (Dementia, Alzheimer Type[Title/Abstract])) OR (Alzheimer Type Dementia[Title/Abstract])) OR (Alzheimer-Type Dementia (ATD[Title/Abstract]))) OR (Alzheimer Type Dementia (ATD[Title/Abstract]))) OR (Dementia, Alzheimer-Type (ATD[Title/Abstract]))) OR (Alzheimer Type Senile Dementia[Title/Abstract])) OR (Primary Senile Degenerative Dementia[Title/Abstract])) OR (Dementia, Primary Senile Degenerative[Title/Abstract])) OR (Alzheimer Sclerosis[Title/Abstract])) OR (Sclerosis, Alzheimer[Title/Abstract])) OR (Alzheimer Syndrome[Title/Abstract])) OR (Alzheimer's Diseases[Title/Abstract])) OR (Alzheimer Diseases[Title/Abstract])) OR (Alzheimers Diseases[Title/Abstract])) OR (Senile Dementia, Alzheimer Type[Title/Abstract])) OR (Acute Confusional Senile Dementia[Title/Abstract])) OR (Senile Dementia, Acute Confusional[Title/Abstract])) OR (Dementia, Presenile[Title/Abstract])) OR (Presenile Dementia[Title/Abstract])) OR (Alzheimer Disease, Late Onset[Title/Abstract])) OR (Late Onset Alzheimer Disease[Title/Abstract])) OR (Alzheimer's Disease, Focal Onset[Title/Abstract])) OR (Focal Onset Alzheimer's Disease[Title/Abstract])) OR (Familial Alzheimer Disease (FAD[Title/Abstract]))) OR (Alzheimer Disease, Familial (FAD[Title/Abstract]))) OR (Familial Alzheimer Diseases (FAD[Title/Abstract]))) OR (Alzheimer Disease, Early Onset[Title/Abstract])) OR (Early Onset Alzheimer Disease[Title/Abstract])) OR (Presenile Alzheimer Dementia[Title/Abstract])) AND ((((((((((((("Acupuncture Therapy"[Mesh]) OR (Acupuncture Treatment[Title/Abstract])) OR (Acupuncture Treatments[Title/Abstract])) OR (Treatment, Acupuncture[Title/Abstract])) OR (Therapy, Acupuncture[Title/Abstract])) OR (Pharmacoacupuncture Treatment[Title/Abstract])) OR (Treatment, Pharmacoacupuncture[Title/Abstract])) OR (Pharmacoacupuncture Therapy[Title/Abstract])) OR (Therapy, Pharmacoacupuncture[Title/Abstract])) OR (Acupotomy[Title/Abstract])) OR (Acupotomies[Title/Abstract])) OR (("Electroacupuncture"[Mesh]) OR (Electro-acupuncture[Title/Abstract]))) OR (("Acupuncture"[Mesh]) OR (Pharmacopuncture[Title/Abstract])))

1. **Cochrane library**

#1 MeSH descriptor: [Alzheimer Disease] explode all trees

#2 (Alzheimer Dementia or Alzheimer Dementias or Dementia, Alzheimer or Alzheimer's Disease or Dementia, Senile or Senile Dementia or Dementia, Alzheimer Type or Alzheimer Type Dementia or Alzheimer-Type Dementia (ATD) or Alzheimer Type Dementia (ATD) or Dementia, Alzheimer-Type (ATD) or Alzheimer Type Senile Dementia or Primary Senile Degenerative Dementia or Dementia, Primary Senile Degenerative or Alzheimer Sclerosis or Sclerosis, Alzheimer or Alzheimer Syndrome or Alzheimer's Diseases or Alzheimer Diseases or Alzheimers Diseases or Senile Dementia, Alzheimer Type or Acute Confusional Senile Dementia or Senile Dementia, Acute Confusional or Dementia, Presenile or Presenile Dementia or Alzheimer Disease, Late Onset or Late Onset Alzheimer Disease or Alzheimer's Disease, Focal Onset or Focal Onset Alzheimer's Disease or Familial Alzheimer Disease (FAD) or Alzheimer Disease, Familial (FAD) or Familial Alzheimer Diseases (FAD) or Alzheimer Disease, Early Onset or Early Onset Alzheimer Disease or Presenile Alzheimer Dementia):ti,ab,kw

#3 #1 or #2

#4 MeSH descriptor: [Acupuncture Therapy] explode all trees

#5 (Acupuncture Treatment or Acupuncture Treatments or Treatment, Acupuncture or Therapy, Acupuncture or Pharmacoacupuncture Treatment or Treatment, Pharmacoacupuncture or Pharmacoacupuncture Therapy or Therapy, Pharmacoacupuncture or Acupotomy or Acupotomies):ti,ab,kw

#6 #4 or #5

#7 MeSH descriptor: [Electroacupuncture] explode all trees

#8 (Electro-acupuncture):ti,ab,kw

#9 #7 or #8

#10 MeSH descriptor: [Acupuncture] explode all trees

#11 (Pharmacopuncture):ti,ab,kw

#12 #10 or #11

#13 #6 or #9 or #12

#14 #3 and #13

1. **Web of Science**

(TS=(Alzheimer Disease) OR AB=(Alzheimer Dementia OR Alzheimer Dementias OR Dementia, Alzheimer OR Alzheimer's Disease OR Dementia, Senile OR Senile Dementia OR Dementia, Alzheimer Type OR Alzheimer Type Dementia OR Alzheimer-Type Dementia ATD OR Alzheimer Type Dementia ATD OR Dementia, Alzheimer-Type ATD OR Alzheimer Type Senile Dementia OR Primary Senile Degenerative Dementia OR Dementia, Primary Senile Degenerative OR Alzheimer Sclerosis OR Sclerosis, Alzheimer OR Alzheimer Syndrome OR Alzheimer's Diseases OR Alzheimer Diseases OR Alzheimers Diseases OR Senile Dementia, Alzheimer Type OR Acute Confusional Senile Dementia OR Senile Dementia, Acute Confusional OR Dementia, Presenile OR Presenile Dementia OR Alzheimer Disease, Late Onset OR Late Onset Alzheimer Disease OR Alzheimer's Disease, Focal Onset OR Focal Onset Alzheimer's Disease OR Familial Alzheimer Disease FAD OR Alzheimer Disease, Familial FAD OR Familial Alzheimer Diseases FAD OR Alzheimer Disease, Early Onset OR Early Onset Alzheimer Disease OR Presenile Alzheimer Dementia)) AND (TS=(Acupuncture Therapy) OR AB=(Acupuncture Treatment OR Acupuncture Treatments OR Treatment, Acupuncture OR Therapy, Acupuncture OR Pharmacoacupuncture Treatment OR Treatment, Pharmacoacupuncture OR Pharmacoacupuncture Therapy OR Therapy, Pharmacoacupuncture OR Acupotomy OR Acupotomies OR "Electroacupuncture" OR Electro-acupuncture OR "Acupuncture" OR Pharmacopuncture))

1. **Embase**

#3. #1 AND #2

#2. 'acupuncture treatment':ab,ti OR 'acupuncture

treatments':ab,ti OR 'treatment,

acupuncture':ab,ti OR 'therapy,

acupuncture':ab,ti OR 'pharmacoacupuncture

treatment':ab,ti OR 'treatment,

pharmacoacupuncture':ab,ti OR

'pharmacoacupuncture therapy':ab,ti OR 'therapy,

pharmacoacupuncture':ab,ti OR acupotomy:ab,ti OR

acupotomies:ab,ti OR 'electroacupuncture':ab,ti

OR 'electro acupuncture':ab,ti OR

'acupuncture':ab,ti OR pharmacopuncture:ab,ti

#1. 'alzheimer dementia':ab,ti OR 'alzheimer

dementias':ab,ti OR 'dementia, alzheimer':ab,ti

OR 'alzheimers disease':ab,ti OR 'dementia,

senile':ab,ti OR 'senile dementia':ab,ti OR

'dementia, alzheimer type':ab,ti OR 'alzheimer

type dementia':ab,ti OR 'alzheimer-type dementia

atd':ab,ti OR 'alzheimer type dementia atd':ab,ti

OR 'dementia, alzheimer-type atd':ab,ti OR

'alzheimer type senile dementia':ab,ti OR

'primary senile degenerative dementia':ab,ti OR

'dementia, primary senile degenerative':ab,ti OR

'alzheimer sclerosis':ab,ti OR 'sclerosis,

alzheimer':ab,ti OR 'alzheimer syndrome':ab,ti OR

'alzheimer diseases':ab,ti OR 'alzheimers

diseases':ab,ti OR 'senile dementia, alzheimer

type':ab,ti OR 'acute confusional senile

dementia':ab,ti OR 'senile dementia, acute

confusional':ab,ti OR 'dementia, presenile':ab,ti

OR 'presenile dementia':ab,ti OR 'alzheimer

disease, late onset':ab,ti OR 'late onset

alzheimer disease':ab,ti OR 'alzheimers disease,

focal onset':ab,ti OR 'focal onset alzheimers

disease':ab,ti OR 'familial alzheimer disease

fad':ab,ti OR 'alzheimer disease, familial

fad':ab,ti OR 'familial alzheimer diseases

fad':ab,ti OR 'alzheimer disease, early

onset':ab,ti OR 'early onset alzheimer

disease':ab,ti OR 'presenile alzheimer

dementia':ab,ti


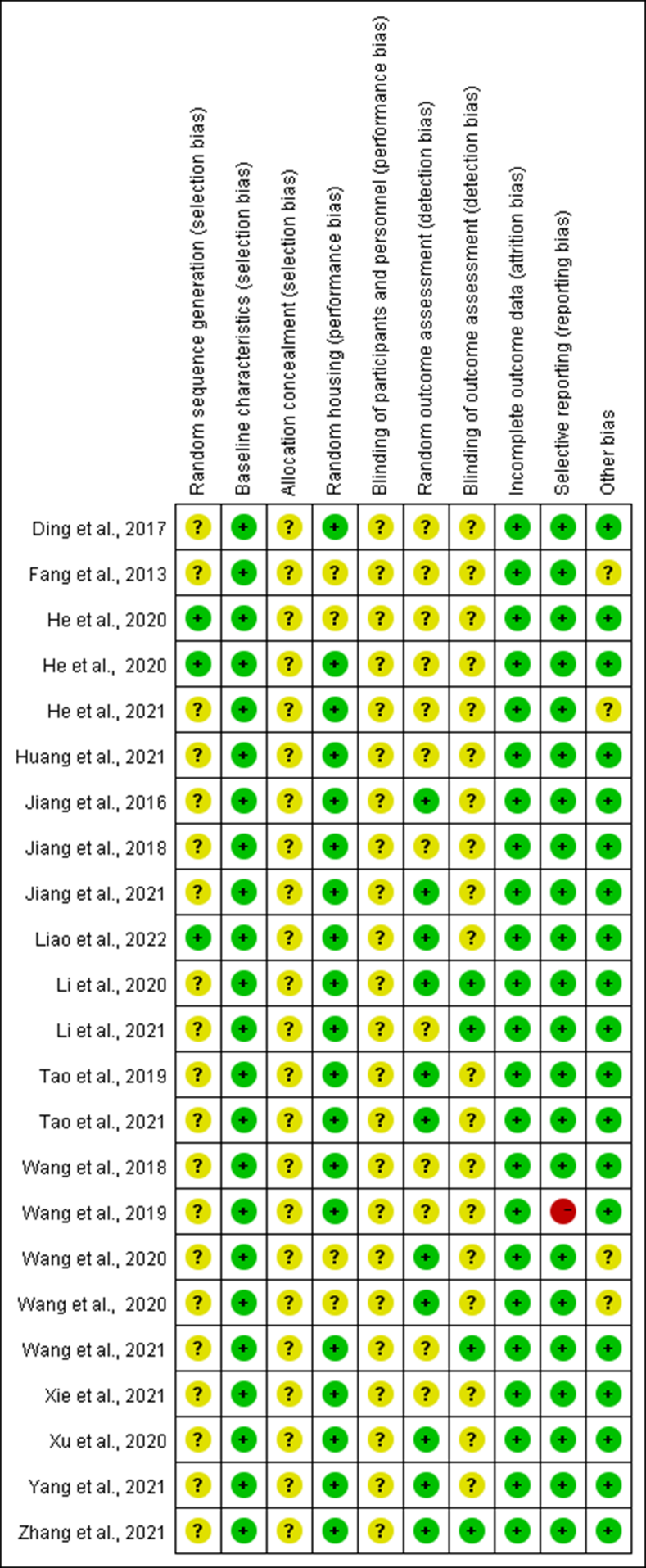


**Supplementary Figure 1.** Risk of bias assessment: the researchers' evaluation of the risk of bias items for each of the included studies.


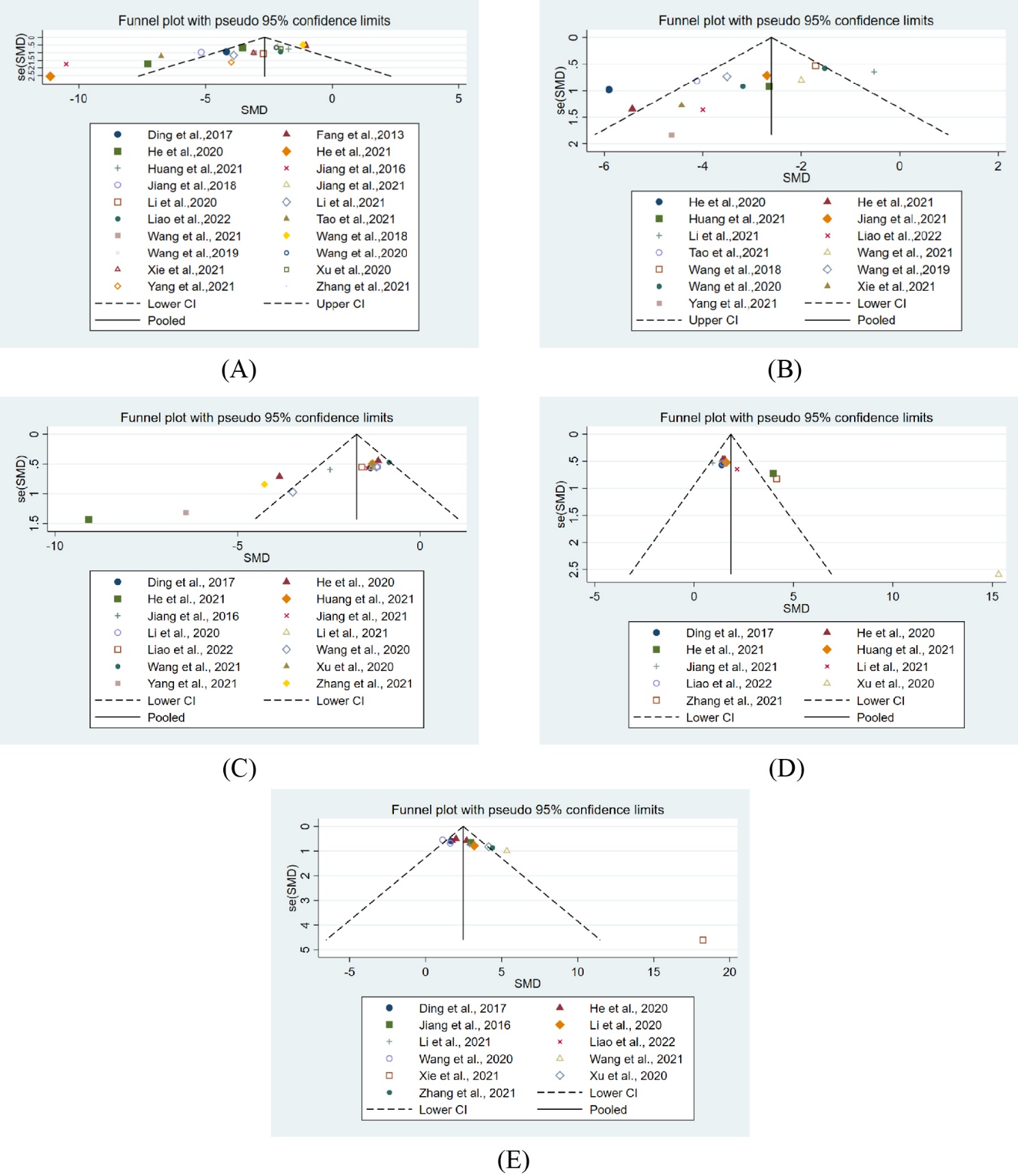


**Supplementary Figure 2.** Funnel plots showing publication bias. (A) IL-1β; (B) TNF-α; (C) escape latency; (D) the duration in platform quadrant; (E) platform crossing number.


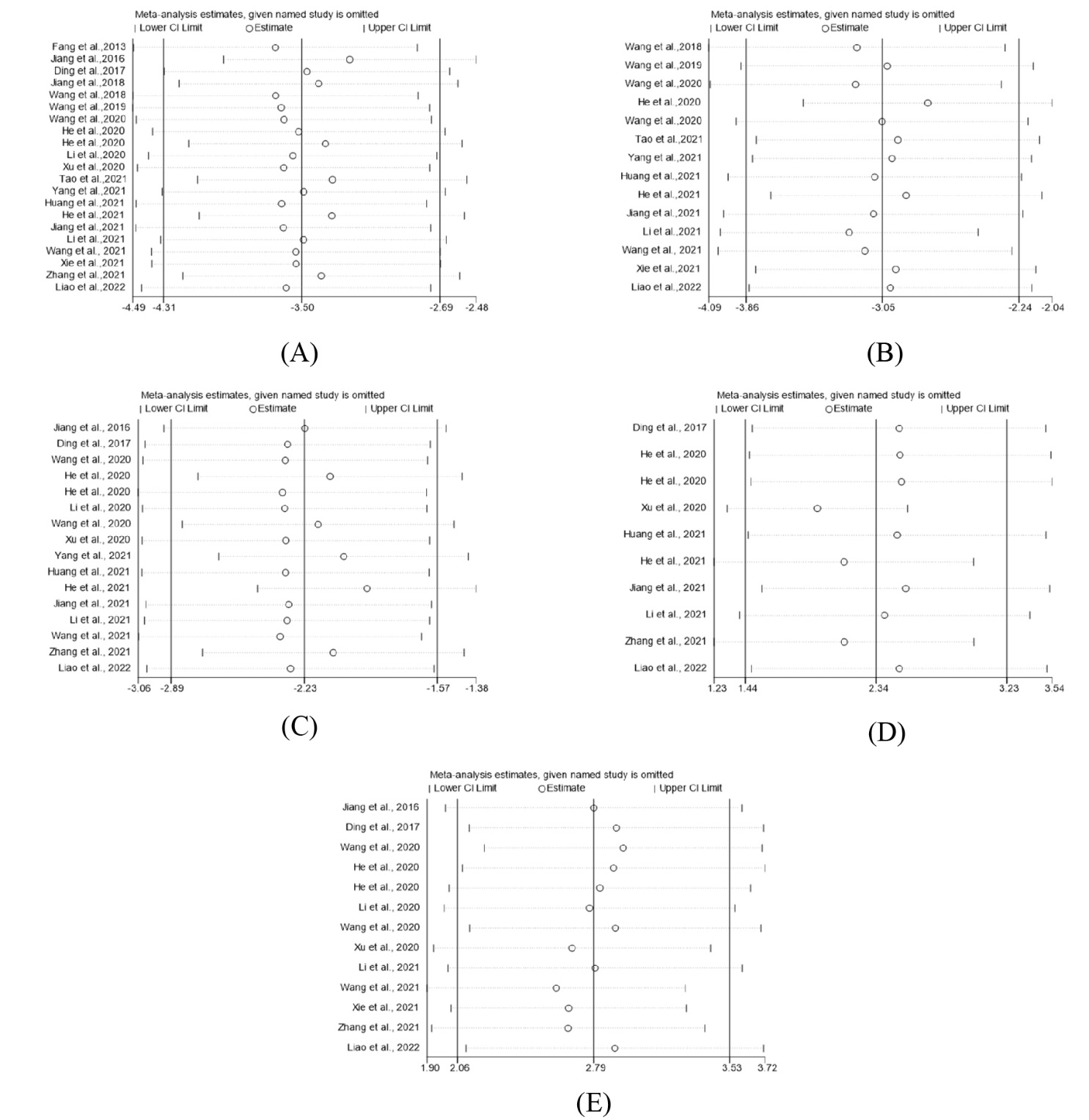


**Supplementary Figure 3.** The sensitivity analysis of included studies. **(A)** IL-1β; **(B)** TNF-α; **(C)** escape latency; **(D)** the duration in platform quadrant; **(E)** platform crossing number.
